# Mesenchymal niche remodeling impairs hematopoiesis via stanniocalcin 1 in acute myeloid leukemia

**DOI:** 10.1172/JCI133187

**Published:** 2020-05-04

**Authors:** Alexander Waclawiczek, Ashley Hamilton, Kevin Rouault-Pierre, Ander Abarrategi, Manuel Garcia Albornoz, Farideh Miraki-Moud, Nourdine Bah, John Gribben, Jude Fitzgibbon, David Taussig, Dominique Bonnet

**Affiliations:** 1 Haematopoietic Stem Cell Laboratory, Francis Crick Institute, London, United Kingdom.; 2 Haemato-Oncology Unit, Royal Marsden Hospital, Institute of Cancer Research, London, United Kingdom.; 3 Bioinformatic Core Facility, Francis Crick Institute, London, United Kingdom.; 4 Haemato-Oncology, Barts Cancer Institute, Queen Mary University of London, London, United Kingdom.

**Keywords:** Hematology, Stem cells, Adult stem cells, Hematopoietic stem cells, Leukemias

## Abstract

Acute myeloid leukemia (AML) disrupts the generation of normal blood cells, predisposing patients to hemorrhage, anemia, and infections. Differentiation and proliferation of residual normal hematopoietic stem and progenitor cells (HSPCs) are impeded in AML-infiltrated bone marrow (BM). The underlying mechanisms and interactions of residual hematopoietic stem cells (HSCs) within the leukemic niche are poorly understood, especially in the human context. To mimic AML infiltration and dissect the cellular crosstalk in human BM, we established humanized ex vivo and in vivo niche models comprising AML cells, normal HSPCs, and mesenchymal stromal cells (MSCs). Both models replicated the suppression of phenotypically defined HSPC differentiation without affecting their viability. As occurs in AML patients, the majority of HSPCs were quiescent and showed enrichment of functional HSCs. HSPC suppression was largely dependent on secreted factors produced by transcriptionally remodeled MSCs. Secretome analysis and functional validation revealed MSC-derived stanniocalcin 1 (STC1) and its transcriptional regulator HIF-1α as limiting factors for HSPC proliferation. Abrogation of either STC1 or HIF-1α alleviated HSPC suppression by AML. This study provides a humanized model to study the crosstalk among HSPCs, leukemia, and their MSC niche, and a molecular mechanism whereby AML impairs normal hematopoiesis by remodeling the mesenchymal niche.

## Graphical abstract

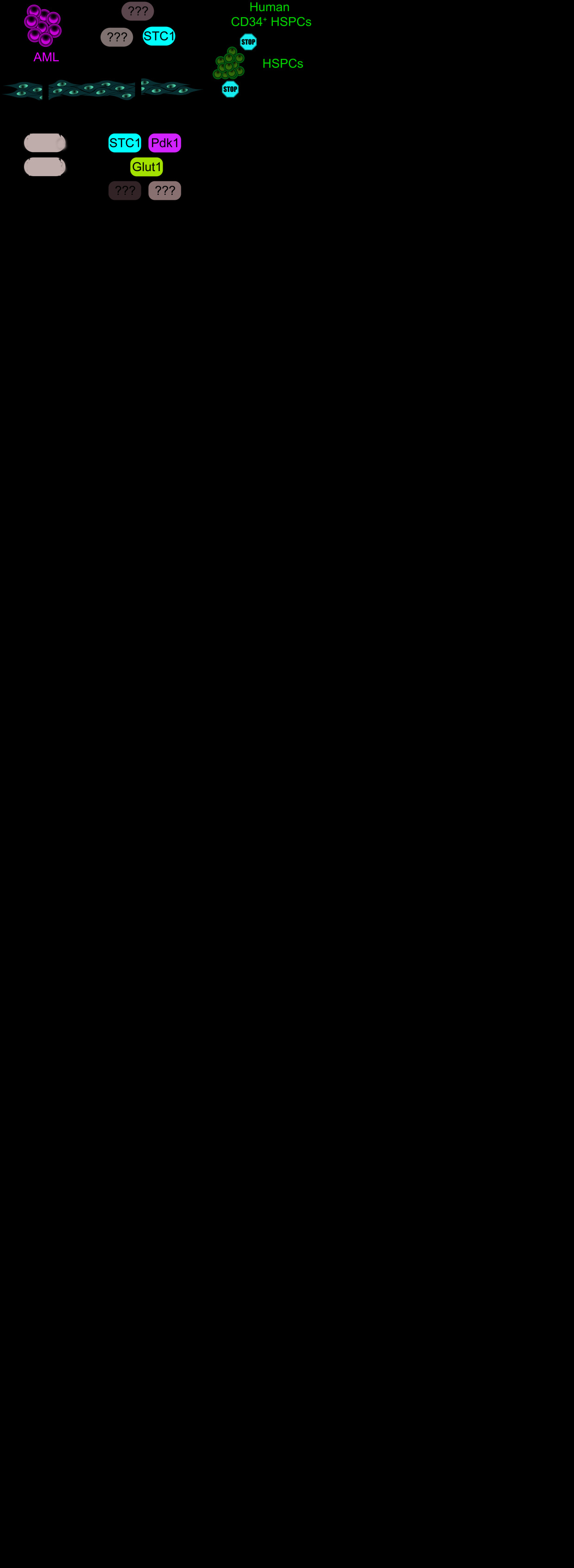

## Introduction

The hematopoietic system is a conglomerate of multiple lineages with the hematopoietic stem cell (HSC) as common origin (1). Under normal circumstances, hematopoiesis is tightly balanced between expansion and quiescence to provide adequate output (2). Extrinsic (3) and intrinsic factors (4) ensure homeostasis but also allow adaptation (5) and recovery (6) to high-demand circumstances such as infections or BM transplantation. In the BM, HSCs reside within a protective niche composed of an interconnected network of hematopoietic and non-hematopoietic cells that guide HSC quiescence, expansion, and homing (3). Within non-hematopoietic cells, mesenchymal stromal cells (MSCs) are an essential niche element (7, 8). MSCs are a well-characterized but heterogeneous population that resides in close proximity to the vasculature (9, 10) and expresses key hematopoietic factors such as stem cell factor (SCF) and stromal cell–derived factor 1 (SDF-1) (11, 12). MSCs can give rise to other BM components of adipo-, osteo-, and chondrogenic lineage (7, 13) and de novo generate HSC- and AML-supportive extramedullary hematopoietic niches (14–16).

Despite having an extensive vascular network, the BM niche is characterized by low oxygen pressure (or hypoxia) (17). HIFs form heterodimers composed of the oxygen-sensitive α-subunit and the ubiquitously expressed β-subunit. HIFs regulate the cellular response to hypoxia partly by binding to specific motifs upstream of hypoxia-responsive genes (18). For human hematopoietic stem and progenitor cells (HSPCs), the hypoxic environment and HIFs are essentially involved in anaerobic metabolism and redox homeostasis to support HSC self-renewal and dormancy (19, 20). Besides HSPCs, several niche components including endothelial cells and MSCs express high levels of HIFs (21–24).

It was shown in mouse AML models that AML cells extensively reorganize the cellular, physical, and transcriptional niche architecture to facilitate their own expansion (25–27) and provide protection from cytotoxic chemotherapy (28). The leukemic BM contains a dysregulated vascular and mesenchymal network with reduced perfusion and oxygen availability (28, 29). In humans, similar niche remodeling may also occur, since MSCs isolated from AML patients are transcriptionally (30, 31), genetically (32–34), and functionally (35, 36) distinct from healthy donor counterparts. MSCs from AML patients downregulate hematopoietic maintenance and homing factors (35) and show adipogenic and osteogenic differentiation (35–37) deficiencies that change the composition of the cellular niche (30). BM failure (reduced production of neutrophils, erythrocytes, and platelets) is almost universal at diagnosis, and contributes to morbidity and mortality by predisposing patients to infections and severe bleeding (38, 39).

Our previous work on AML patient samples and patient-derived xenograft (PDX) models demonstrated an enrichment of HSCs at the expense of less-primitive progenitors among the remaining normal hematopoietic cells in the BM (40). Consistent with this, most younger patients achieve rapid reversal of marrow failure on attainment of remission following intensive chemotherapy. AML-induced cytopenias might be expected to drive residual HSPCs into cell cycle through feedback, but HSPCs are predominantly quiescent (40–43) and express aberrant levels of the negative cell cycle regulators *p19*, *p21*, and *Egr1–3* (42). These observations imply that BM failure is not a consequence of HSC depletion but rather may involve dysregulation of cell cycle activation and differentiation.

The molecular mechanisms governing the suppression of normal hematopoiesis are poorly understood but could provide insight into HSC regulation. Several lines of evidence from murine studies suggest an indirect mechanism via a dysregulated BM niche (30, 35), but direct evidence from a fully humanized model system is lacking. Outside the direct leukemic context, individual factors such as cytokines (43, 44), exosomes (45, 46), and AML patient MSCs (35) have been investigated, but only as isolated components and not as part of a holistic approach. Additionally, studies on MSCs from AML patients usually required extensive ex vivo culture, outside the leukemia context, which could modify their transcriptomic signatures (47).

In this study, we modified 2 established fully humanized hematopoietic niche systems on the basis of MSC coculture to investigate the multidirectional crosstalk among AML, HSPCs, and the microenvironment. HSPCs recapitulated reversible proliferation and differentiation inhibition by AML cells, which was linked to transcriptional and secretome alterations of the stromal niche. Further investigation and functional validation identified stanniocalcin 1 (STC1) and its transcriptional regulator HIF-1α as niche-specific negative regulators of HSPC proliferation.

## Results

### AML inhibits normal CD34^+^ expansion in an ex vivo humanized niche model.

**Figure 1:**
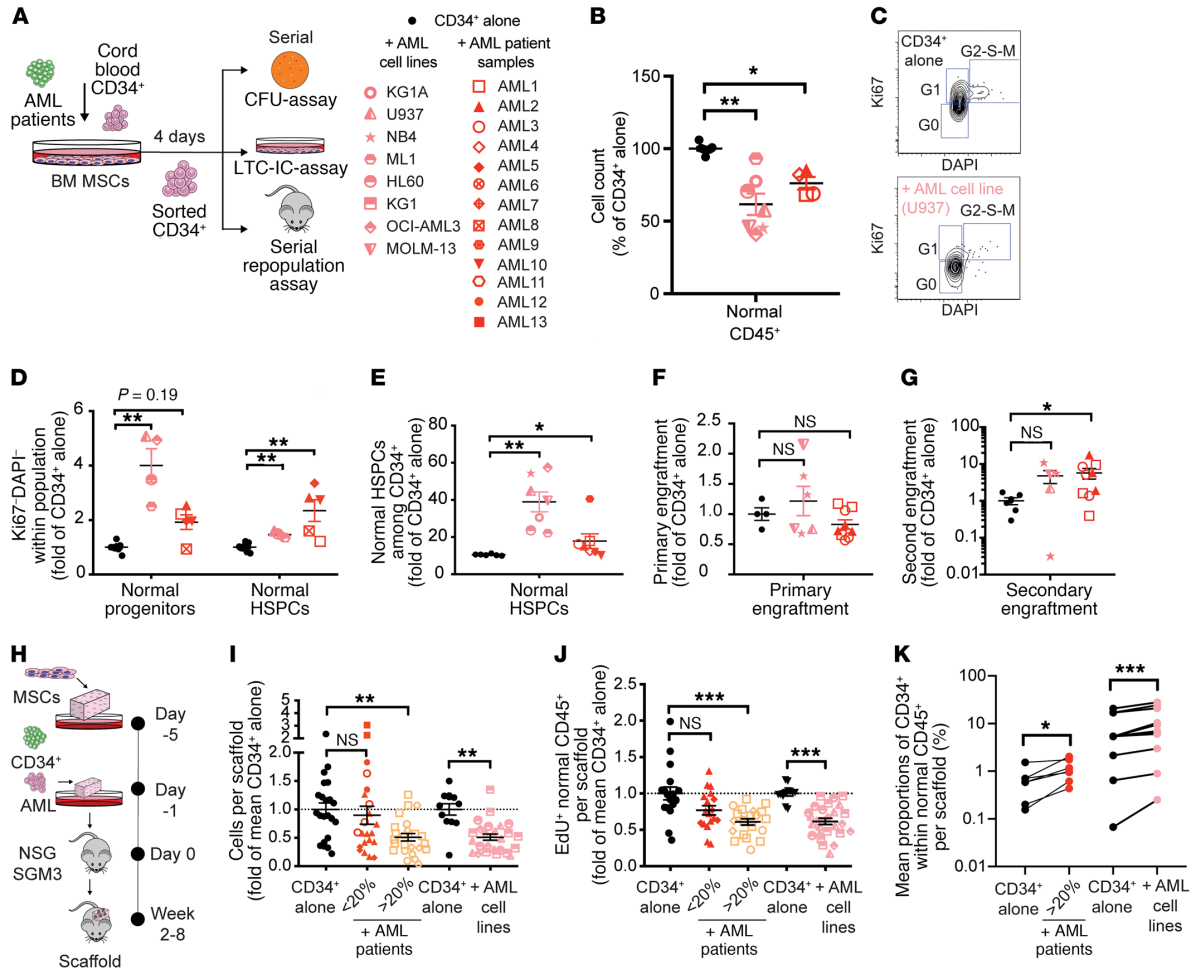
AML induces quiescence and prevents differentiation in normal HSPCs. (**A**–**G**) CD34^+^ cells cocultured with MSCs alone (CD34^+^ alone) (*n* = 4–7) or +AML cell lines (*n* = 3–7) or +AML primary patient samples (*n* = 3–7). After 4 days of coculture, CD34^+^ cells were plated for CFU or LTC-IC assays or implanted into NSG mice. (**B**) Cell counts of total non-leukemic hematopoietic cells. AML patient samples: AML1–4. (**C**) Representative FACS plots of cell cycle analysis of CD34^+^ cells based on DAPI and Ki-67 staining. (**D**) Quiescent (Ki-67^–^DAPI^–^) cells within normal progenitors (CD34^+^CD38^+^) and HSPCs (CD34^+^CD38^–^). AML1, -2, -5, -8, and -9. (**E**) Proportions of normal HSPCs within CD34^+^ cells. AML1-5, -8, and -9. (**F**) Engraftment in primary NSG recipients. Three independent experiments with 1–7 mice/group per experiment. (**G**) Secondary recipients. AML1–3. Three independent experiments with 2–4 mice/group per experiment. (**H**–**K**) Collagen scaffolds seeded with MSCs were injected with CB CD34^+^ cells alone or +GFP^+^ AML cell lines (*n* = 4) and transplanted into NSG-SGM3 recipients. In the case of AML patient samples (*n* = 5–8), the CB CD34^+^ cells were either HLA-A2 mismatched or transduced to express GFP. Scaffold retrieval: 2–3 weeks (+AML cell lines) or 5–8 weeks (+AML patient samples). EdU i.p. injection: 16 hours prior to scaffold retrieval. (**I**) Non-leukemic human CD45^+^ hematopoietic cells per scaffold. AML1–5 and 11–13. Twelve to 26 scaffolds in 6–10 mice/group. (**J**) EdU incorporation in non-leukemic human CD45^+^ hematopoietic cells. AML1–5. Twelve to 26 scaffolds in 5–7 mice/group. (**K**) Proportions of CD34^+^ HSPCs within non-leukemic human CD45^+^ hematopoietic cells. Five to 11 mice/group. AML1, -3, and -4. Each AML cell line or patient sample is represented as a unique symbol. CD34^+^ cells alone were used as control and for normalization. Data are presented as mean ± SEM. *P < 0.05, **P < 0.01, ***P < 0.001 by Mann-Whitney *U* test (**B** and **D**–**G**), Kruskal-Wallis with Dunn’s (**I** and **J**), and Wilcoxon’s matched-pairs signed-rank test (**K**).

Cytopenias are frequent symptoms of AML. Nonetheless, AML does not deplete normal HSPCs but rather suppresses normal differentiation and proliferation (40). Consistent with this, when we compared the expansion of cord blood (CB) CD34^+^ cells cultured with healthy donor MSCs alone (CD34^+^-alone) or together with AML cell lines (+AML cell lines) for 4 days (Figure 1A), we observed that AML cell lines decreased the retrieval of normal hematopoietic cells by 38% ± 19.5% (Figure 1B). This observation was confirmed with primary patient samples (+AML patient samples) showing a reduction in human normal CD45^+^ cells of 23% ± 8.7% (Figure 1B). The viability of normal HSPCs did not differ between control and AML conditions and was generally greater than 96% and 89% for cell lines and AML samples, respectively (data not shown).

CD34^+^ cells freshly isolated from CB are exclusively in the G_0_/G_1_ phase of the cell cycle but enter S/G_2_/M phases in culture after 24–48 hours (48). Cell cycle analysis based on Ki-67 expression and DNA content (DAPI) (exemplified in Figure 1C) revealed an enrichment of quiescent cells among normal HSPCs from +AML groups (3.3-fold and 1.6-fold increase compared with CD34^+^-alone for +AML cell lines and +AML patient samples, respectively) (Figure 1D). Similarly, pulse-chase experiments with the semipermeable dye CellTrace violet (CTV) (exemplified in Supplemental Figure 1A; supplemental material available online with this article; https://doi.org/10.1172/JCI133187DS1) showed a significant increase in CTV^bright^ progenitors and HSPCs in groups with AML cells, indicating that these cells undergo fewer divisions (Supplemental Figure 1, B and C). These data suggest that ex vivo, AML suppresses CD34^+^ expansion by promoting quiescence but not apoptosis.

### HSPCs from AML cocultures are enriched in primitive hematopoietic cells.

In parallel to the proliferative deficits, the remaining CD34^+^ cells in the +AML groups adopted a more immature HSPC phenotype (CD34^+^CD38^–^) than CD34^+^ cells alone, indicating a potential differentiation defect (Figure 1E). However, downregulation of CD38 in normal progenitors in culture can contaminate HSPCs, which could have obscured our interpretation (49). We thus performed functional assays including serial CFU, long-term culture initiating cell (LTC-IC), and serial transplantation in sublethally irradiated NOD/SCID/IL2Rγ^null^ mice on sorted CD34^+^ cells after 4 days of coculture with or without AML (Figure 1A). Of note, in preliminary experiments, AML cell lines contaminated the sorted CD34^+^ cells and compromised subsequent experiments. Therefore, for AML cell lines, we separated MSCs from CD34^+^ cells via a 0.4-μm Transwell insert. Despite the fact that there was no direct MSC-AML cell line contact, this culture system mimicked the suppression of normal HSPCs generated via direct contact conditions. In contrast, as primary AML patient samples contained fewer leukemia-initiating cells, we could coculture MSCs and AML cells in direct contact without observing any contamination from AML cells after sorting CD34^+^ cells and assessing their ex vivo or in vivo functionalities.

We observed an increase in the number of primary and secondary colony-forming cells (CFCs) in CD34^+^ cells that had been cultured in the presence of AML (both cell lines and primary AML) (Supplemental Figure 1, D and E). Similarly, we saw a 2-fold increase in LTC-IC frequency in the presence of AML (Supplemental Figure 1F), suggesting an enrichment of primitive hematopoietic cells. In vivo analysis showed comparable levels of human hematopoietic engraftment in mice sacrificed after 12 weeks (Figure 1F). However, when primary engrafted human CD45^+^ cells from +AML patient samples were transplanted into secondary mice, they generated significantly more engraftment than equivalent numbers of human CD45^+^ cells from the CD34^+^-alone group. A similar trend was established for +AML cell lines, but the difference was not statistically significant (Figure 1G). These data suggest that hematopoietic cells in the presence of AML are enriched in functional HSPCs, and their proliferative and differentiation potential can be restored in the absence of AML.

### AML inhibits normal CD34^+^ cell engraftment in an in vivo humanized niche model.

To validate the results from the humanized ex vivo model, we made use of our recently developed humanized niche model (14). Briefly, human MSCs (hMSCs) were seeded in partially dehydrated gelatin-based porous scaffolds and expanded for 3 days before injection of CB CD34^+^ cells alone or with AML cell lines or AML patient samples, and maintained in NSG-S mice for up to 8 weeks (Figure 1H). To distinguish normal human hematopoietic cells (hCD45^+^) from leukemic cells, we either lentivirally transduced CD34^+^ cells to express GFP (Supplemental Figure 1G) or paired CB donor and AML patient samples with mismatching HLA-A expression (data not shown). Although the normal/leukemic chimerism was overwhelmingly leukemic across all AML cell lines, intersample differences in AML patients prompted us to stratify AML into 2 groups based on the percentage of human engraftment (normal and leukemic) (<20% and >20%). Retrieval of nonleukemic hCD45^+^ cells from scaffolds with leukemic cell lines as well as primary samples with more than 20% engraftment was significantly reduced (Figure 1I). The proportions of viable cells within normal hCD45^+^ cells were similar in the CD34^+^-alone and +AML groups (Supplemental Figure 1H); but cell cycle activity, measured by incorporation of 5-ethynyl-2′-deoxyuridine (EdU) into normal hCD45^+^ cells, was diminished by 38% in scaffolds injected with AML cell lines and 42% with highly engrafted AML patient samples (Figure 1J). Additionally, both normal hCD45^+^ and CD34^+^ cells from AML cell line scaffolds retained 2-fold more CTV^+^ cells than their CD34^+^-alone counterparts (Supplemental Figure 1I). As in our ex vivo system, we observed a significant enrichment of phenotypic CD34^+^ cells within nonleukemic CD45^+^ cells in scaffolds with highly engrafted AML patient samples and AML cell lines (Figure 1K). Enrichment of primitive cells was confirmed by the increased frequency of nonleukemic primary CFU from leukemic scaffolds (Supplemental Figure 1J). These data demonstrate that AML cells inhibit engraftment of CD34^+^ cells in humanized niches by suppressing their cell cycle activity.

### AML suppresses HSPC proliferation by remodeling the mesenchymal niche.

**Figure 2:**
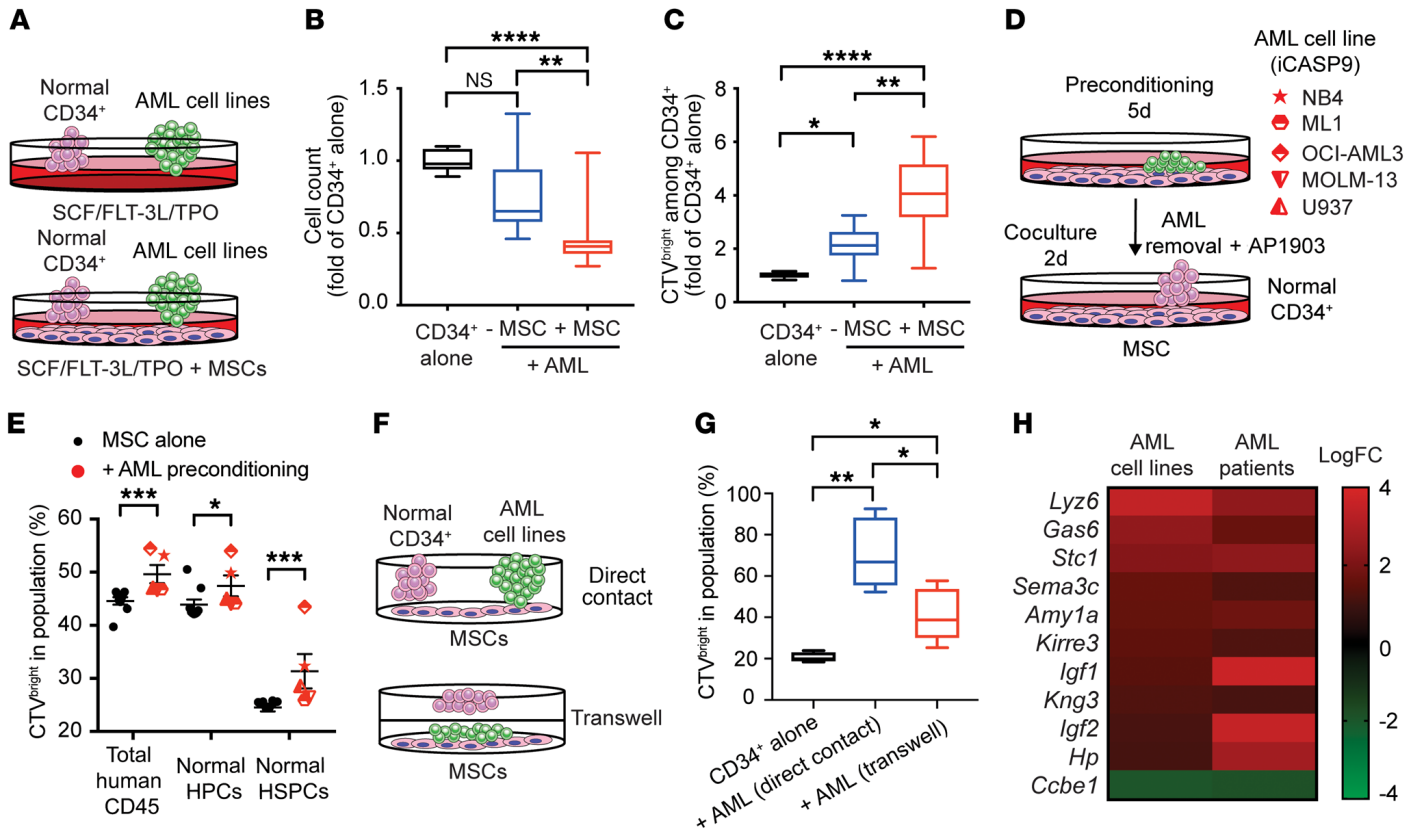
AML induces HSPC quiescence via mesenchymal niche–secreted factors. (**A**–**C**) CD34^+^ cells were cultured alone or with AML cell lines with 100 ng/mL SCF/FLT-3L and 20 ng/mL TPO + 10% FBS with (+) and without (–) MSCs. CD34^+^ cells alone were used as control and reference for normalization. Cell counts of CD34^+^ cells after 4 days (**B**) and CTV^bright^ cells among CD34^+^ cells (**C**). (**D** and **E**) MSCs were cultured alone (*n* = 6) or cocultured with AML cell lines expressing iCASP9 (*n* = 5) for 5 days. AP1903 (5 nM) was added on day 4, inducing apoptosis and allowing the removal of AML. CB CD34^+^ cells were then added in fresh medium to the preconditioned MSCs for 48 hours. (**E**) Percentage of CTV^bright^ cells retrieved after 48 hours. (**F**) CD34^+^ cells cocultured with MSCs and AML cell lines (*n* = 3) directly or separated from MSCs and AML via a 0.4-μm Transwell insert. (**G**) CTV^bright^ cells among CD34^+^ cells. (**H**) Gene expression analysis of MS-5 cells after coculture for 7 days with AML cell lines (*n* = 7), AML patient samples (*n* = 2), or normal CD34^+^ cells (*n* = 3) as control. Heatmap of shared upregulated secreted factors with logarithmic fold change (logFC) > 1. FC, fold change. Data are presented as mean ± SEM, with each AML cell line or patient sample as a unique symbol (**E**) or as box-and-whisker plots, with bounds from 25th to 75th percentile, median line, and whiskers ranging from smallest to largest values of 4 measurements from CD34^+^ alone or +AML cell lines (*n* = 3–4) (**B**, **C**, and **G**). *P < 0.05,**P < 0.01, ***P < 0.001, ****P < 0.0001 by Kruskal-Wallis test with Dunn’s test (**B**, **C**, and **G**) and Mann-Whitney *U* test (**E**).

Extensive remodeling of the BM niche by AML has been reported in several ex vivo and in vivo model systems (27, 28, 36). We hypothesized that indirect interactions via the mesenchymal niche may also form the basis for HSPC suppression in our model. First, we cocultured normal CD34^+^ cells with AML cell lines alone or with MSCs under conditions that permit feeder-free maintenance (50) to assess the impact of direct and indirect interactions (Figure 2A). Although direct interactions reduced CD45 retrieval by 25% and induced a 2-fold increase in CTV^bright^ cells among CD34^+^ cells, the addition of MSCs further reduced CD45 retrieval to 45% and increased the CTV^bright^ population by 3.2-fold (Figure 2, B and C, and Supplemental Figure 2A). To eliminate AML-MSC interactions as the sole cause, we first preconditioned MSCs with AML cell lines (transduced with an inducible caspase-9 [iCASP9] suicide gene) for 5 days and then cocultured them with normal CD34 after removal of the AML cell lines using AP1903 to kill specifically all AML cell lines (ref. 51, Figure 2D, and Supplemental Figure 2B). The conditioning of MSCs by AML was sufficient to induce a significant increase in the retention of CTV among CD34^+^ cells after 2 days coculture (Figure 2E). These data imply that the remodeling of the mesenchymal niche by AML reduces HSPC proliferation. Nevertheless, at this stage, we did not know whether the altered MSC was inhibiting HSPC via direct contact. We thus cocultured MSCs plus AML and added CTV-stained CD34^+^cells to this coculture either directly or separated by a Transwell (see Figure 2F). After 48 hours, we observed that AML, in both direct or Transwell conditions, increased the retention of CTV among CD34^+^ cells, indicating that both contact and secreted factors from altered MSCs inhibited HSPC proliferation (Figure 2G).

### Secretome analysis identifies STC1.

To identify the niche-derived regulators of HSPC proliferation, we performed microarray analysis and compared the transcriptome of MS-5 MSCs cocultured with AML patient samples or cell lines against that of MS-5 cells cocultured with normal CD34^+^ cells. Since the proliferation of CD34^+^ cells remained significantly suppressed even when they were separated from AML and MSCs via a Transwell insert (see Figure 2G), we searched for putative secreted factors (Figure 2H and Supplemental Figure 2C). Several known regulators of HSPCs were upregulated in MS-5 cells cocultured with AML, such as AXL ligand, *GAS6* (52), metalloproteinase *KIRRE3* (53), and *Igf1* and -*2* (54). We also found upregulation of *STC1*. As *STC1* expression has been notated in several solid cancers as an adverse prognostic marker, we went on to investigate its role here.

### STC1 is a negative regulator of HSPC proliferation.

**Figure 3:**
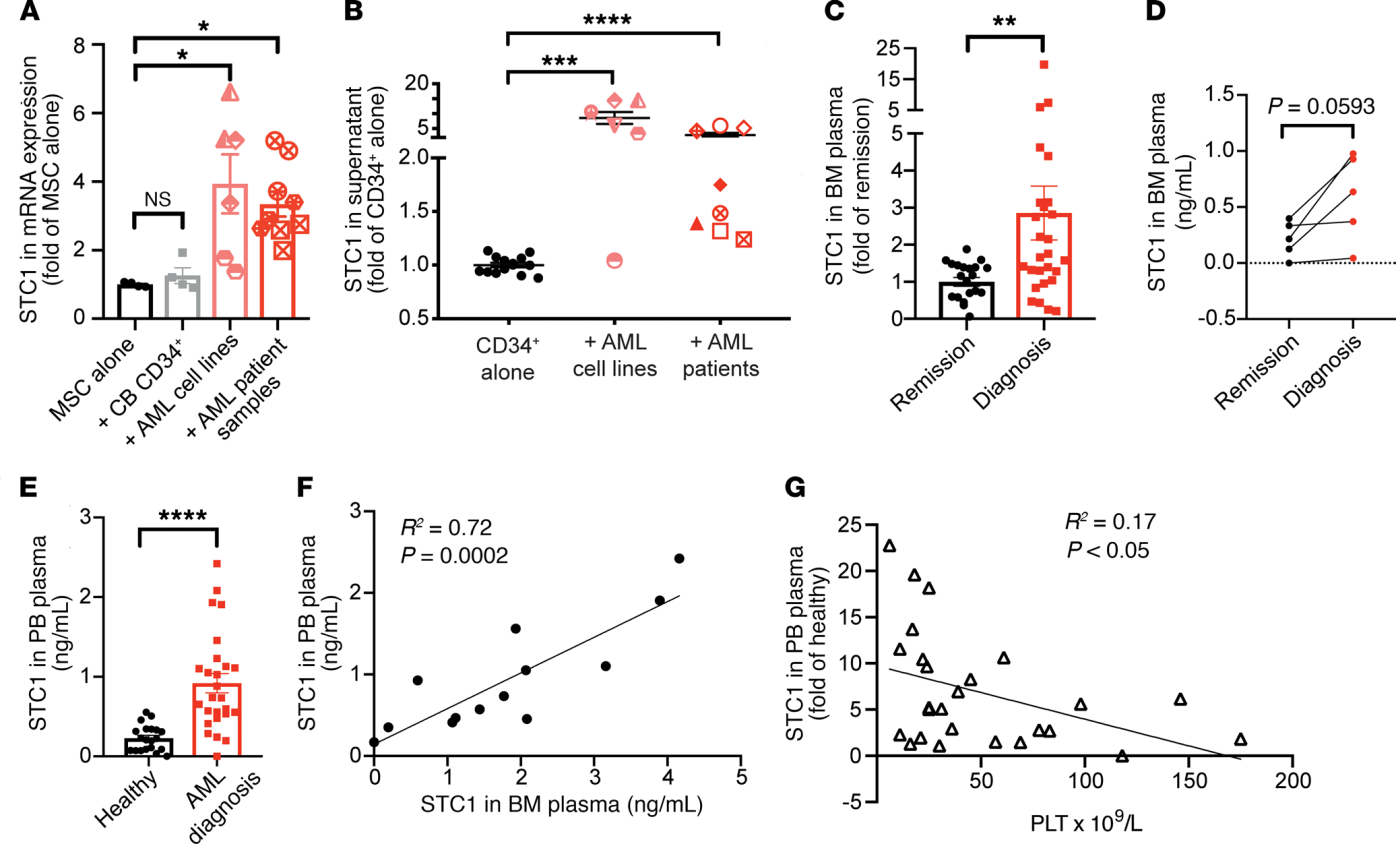
MSC-secreted STC1 is upregulated in AML patient plasma. (**A**) mRNA expression of *STC1* in MSCs cultured alone (*n* = 4), +CB CD34^+^ cells (*n* = 4), or +AML cell lines (*n* = 3)/patient samples (*n* = 3) for 5 days; measured by quantitative PCR (qPCR) and normalized to MSCs cultured alone (*n* = 4). AML patient samples: AML6, -8, and -9. (**B**) STC1 protein in supernatant of MSCs cocultured with CD34^+^ cells, +AML cell lines (*n* = 7)/patient samples (*n* = 8); measured by ELISA and normalized to MSCs + CD34^+^ cells (CD34^+^ alone). AML1–5, -8, -10, and -11. STC1 protein was measured by ELISA (**C**) in AML patient BM plasma from nonmatched diagnosis (*n* = 27) and remission (*n* = 21) samples and normalized to remission and (**D**) from 5 matched diagnosis/remission samples. (**E**) STC1 protein in AML patient PB plasma at diagnosis (*n* = 26) and healthy donors (*n* = 20). (**F**) Correlation of STC1 protein concentration between BM and PB plasma (*n* = 13). (**G**) Correlation of platelet (PLT) count in PB and STC1 protein in PB plasma from AML patients at diagnosis (*n* = 26). Data are presented as mean ± SEM, with each patient sample as a dot (**C**–**G**) or unique symbol (**A** and **B**). *P < 0.05, **P < 0.01, ***P < 0.001, ****P < 0.0001 by Kruskal-Wallis test with Dunn’s test (**A** and **B**), Mann-Whitney *U* test (**C** and **E**), Wilcoxon’s matched-pairs signed-rank test (**D**), and Pearson’s correlation and linear regression test (**F** and **G**).

We confirmed a 3- to 4-fold increase in *STC1* mRNA expression in human primary MSCs cocultured with AML (cell lines or patient samples) but not with CB CD34^+^ cells (Figure 3A). STC1 levels in the supernatant of MSCs + AML groups ranged between 0.5 and 1.15 ng/mL, showing an up to 10-fold increase compared with MSCs + CD34^+^ cells alone (0.035–0.4 ng/mL) (Figure 3B). *STC1* mRNA expression was not detected in normal or malignant hematopoietic cells, implying that STC1 in cell culture supernatant was stromal in origin (data not shown).

To validate the presence of STC1 in primary AML samples, we determined the level of STC1 in BM plasma of 27 primary AML samples at diagnosis and 21 samples at the time of remission (Figure 3C). The level of STC1 was 3-fold greater in the BM plasma at diagnosis compared with remission. From 5 patients, we obtained BM plasma at both diagnosis and remission, and saw a reduction in STC1 in all matched remission samples (Figure 3D). Similarly, the level of STC1 detected in peripheral blood (PB) of AML patients at diagnosis was significantly higher than in healthy controls (0.92 ng/mL ± 0.62 AML versus 0.23 ± 0.16 ng/mL control) (Figure 3E). We further correlated the level of STC1 in BM to PB in the same patients, indicating that measurement of STC1 in the blood could be an alternative to BM aspiration (Figure 3F). Last, we found a significant inverse correlation between the level of STC1 and the platelet count (Figure 3G). These data demonstrate that STC1 is elevated in primary AML compared with healthy control samples and reinforce our ex vivo results.

**Figure 4:**
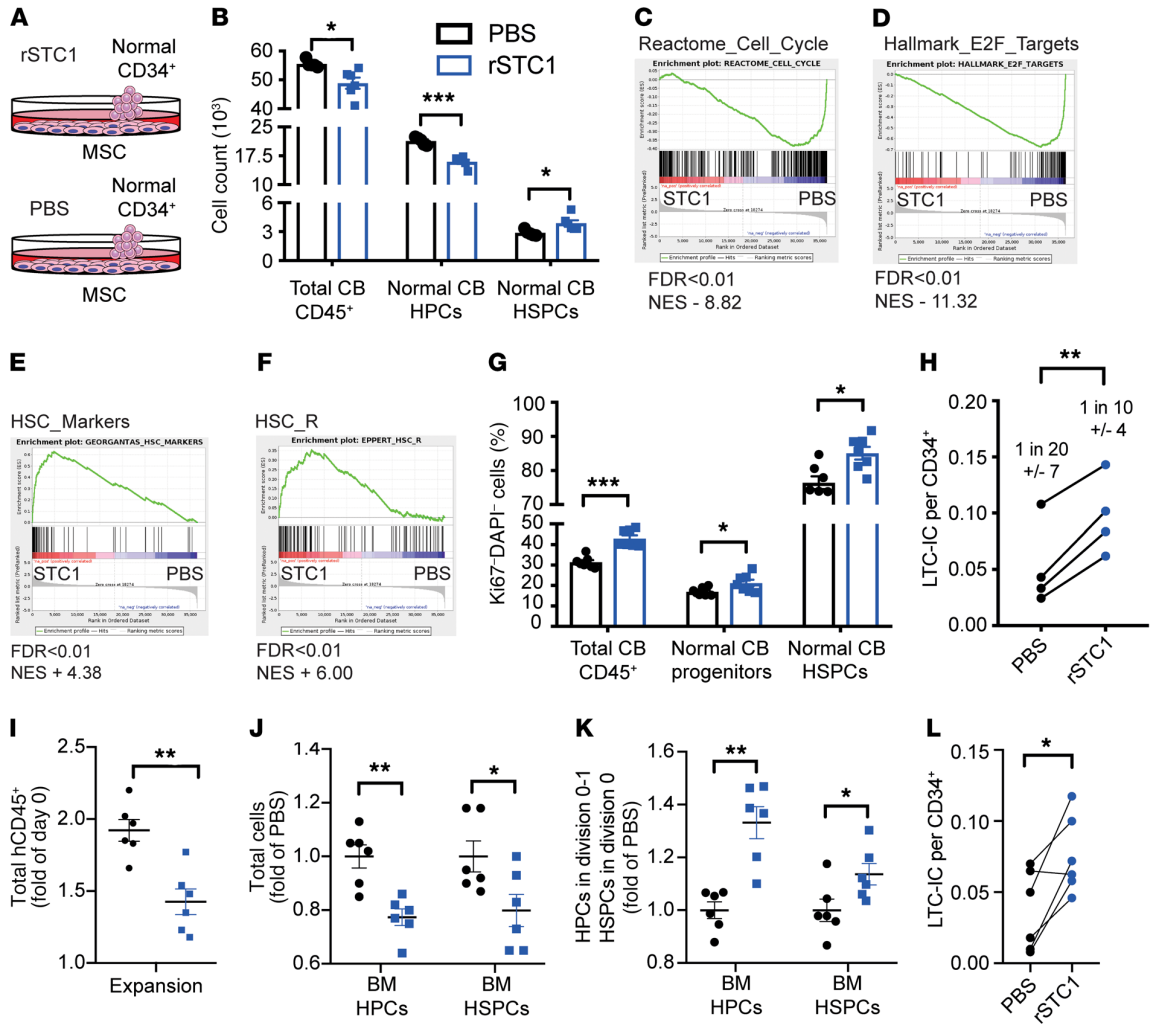
STC1 induces reduction of CB and BM HSPC proliferation ex vivo. (**A** and **B**) MSCs were cocultured with CB CD34^+^ cells and supplemented with rSTC1 or PBS (vehicle control) for 5 days. (**B**) Quantification of cell count. (**C**–**F**) Results of GSEA of HSPCs (CD34^+^CD38^–^) cocultured for 48 hours with MSCs and supplemented with rSTC1 or PBS (vehicle control) (*n* = 3). (**G**) Proportions of Ki-67^–^ quiescent cells in CB CD34^+^ cells treated with rSTC1 and PBS. (**H**) LTC-IC frequency in sorted CD34^+^ cells. (**I**–**L**) MSCs were cocultured with BM CD34^+^ from 2 donors and supplemented with rSTC1 or PBS (vehicle control) for 5 days. (**I**) Total expansion of hCD45^+^ cells relative to day 0. Quantification of (**J**) HPCs and HSPCs normalized to PBS on day 5 and (**K**) proportions of HPCs/HSPCs that had undergone 0–1 divisions normalized to PBS. (**L**) LTC-IC frequency per CD34^+^ cells. Data are presented as mean ± SEM, *n* = 4–7. *P < 0.05, **P < 0.01, ***P < 0.001 by 2-tailed Student’s *t* test. NES, normalized enrichment score.

To further evaluate the role of STC1 in normal HSPCs, we supplemented recombinant STC1 (rSTC1) in the CB-derived CD34^+^ cell–MSC coculture (see Figure 4A). rSTC1 facilitated the maintenance of primitive HSPCs at the expense of progenitors and total hematopoietic cells (Figure 4B) without affecting viability (Supplemental Figure 3A). Gene set enrichment analysis (GSEA) of RNA-Seq from CD34^+^CD38^–^ HSPCs treated with rSTC1 for 48 hours showed a significant reduction in expression of cell cycle target genes including the known HSC cell cycle regulators Myc (55) and E2F (56) and mTOR targets (ref. 57 and Figure 4, C and D), and showed an enrichment of HSC gene sets (Figure 4, E and F). In agreement with the GSEA, we observed that rSTC1 supplementation increased the proportions of quiescent cells, especially among HSPCs (Figure 4G), and led to an increase in CFCs (both primary and secondary) (Supplemental Figure 3, B and C) and a higher frequency of LTC-ICs among re-sorted CD34^+^ cells (Figure 4H). These data suggest that rSTC1 can mimic the growth-inhibitory effects of AML on HSPCs ex vivo. Surprisingly, AML cell coculture with MSCs supplemented with rSTC1 did not affect the proliferation of AML cells (Supplemental Figure 3D).

To ascertain that the effect of STC1 was not limited to CB HSPCs, we repeated the experiment with healthy adult BM CD34^+^ cells. rSTC1 significantly reduced the number of hCD45^+^ cells and progenitors produced (Figure 4, I and J). Nevertheless, rSTC1 supplementation increased proportions of nondivided hematopoietic progenitor cells (HPCs) and HSPCs compared with control (Figure 4K) and increased the number of secondary CFCs (Supplemental Figure 3E) and LTC-IC frequency (Figure 4L).

**Figure 5:**
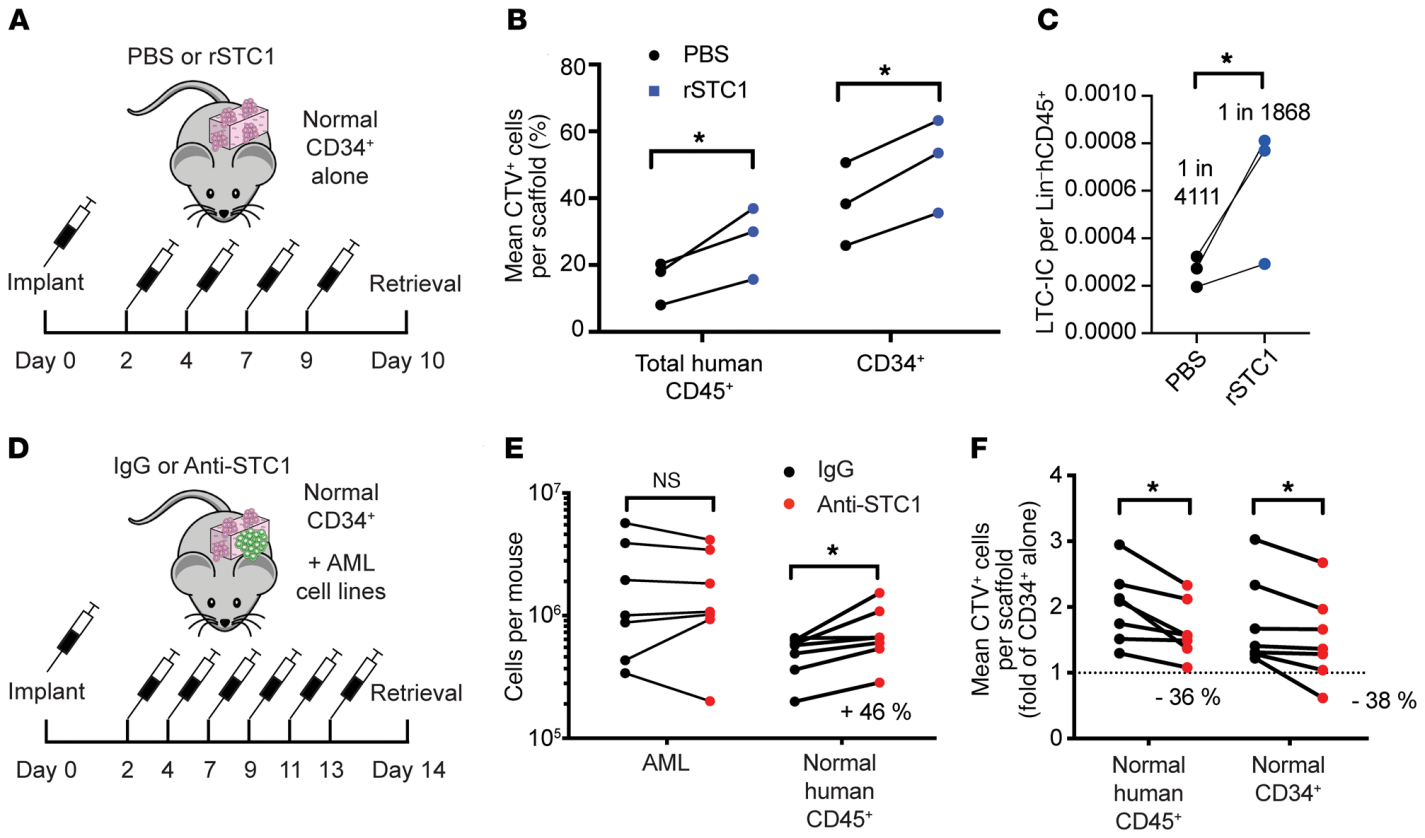
STC1 induces reduction of HSPC proliferation in AML in vivo. (**A**–**C**) MSC scaffolds with normal CD34^+^ cells were implanted into NSG-S mice. rSTC1 was injected every other day for 10 days starting on day 2 after implantation. Three mice/group with 4 scaffolds per mouse. (**B**) CTV^+^ cells among total human CD45^+^ and CD34^+^ cells. (**C**) LTC-IC frequency of sorted human CD45^+^lineage^–^ cells. (**D**–**F**) MSC scaffolds with normal CD34^+^ cells and AML cell lines (U937, OCI-AML3) were implanted into NSG-S mice. IgG or anti-STC1 antibody was injected subcutaneously every other day for 2 weeks. Seven mice/group with 2–4 scaffolds per mouse. (**E**) Absolute cell counts of AML and normal human CD45^+^ cells per mouse. (**F**) CTV^bright^ cells among CD34^+^ cells and normalized to CD34^+^ cells alone. Each dot represents data from 2–6 pooled scaffolds implanted in 1 recipient. *P < 0.05 by 2-tailed paired Student’s *t* test (**B**, **C**, and **F**) or Wilcoxon’s matched-pairs signed-rank test (**E**).

Finally, to validate that STC1 could mimic the effect of AML on HSPCs in vivo, we implanted NSG-S mice with scaffolds containing MSCs with CD34^+^ cells and subcutaneously injected rSTC1 every other day (Figure 5A). After 10 days of treatment, rSTC1-treated scaffolds contained a significantly lower number of total human CD45^+^ cells (Supplemental Figure 4A). Pulse-chase experiments with CTV showed an enrichment of CTV^+^ cells among both total CD45^+^ and CD34^+^ cells (Figure 5B), and in line with our ex vivo results, sorted human lineage^–^CD45^+^ cells contained a higher frequency of LTC-ICs (Figure 5C).

### STC1 neutralization partially rescues HSPC proliferation and engraftment.

To test whether STC1 at endogenous levels has HSPC-regulating effects, we blocked STC1 with a polyclonal antibody ex vivo (Supplemental Figure 4B). STC1 neutralization was associated with a significant increase in cell division among normal CD34^+^ cells cocultured with AML (Supplemental Figure 4, C and D).

Next, to test whether STC1 secretion was involved in reducing normal engraftment in the leukemic setting, we implanted into NSG-S mice scaffolds containing MSC/CD34^+^ cells and AML. As neutralizing experiments consume large quantities of antibody and primary AML samples take longer to engraft, thus extending the period for which anti-STC1 is needed, we restricted our analysis to 2 AML cell lines: U937 and OCI-AML3.

Shortly after implantation, we injected STC1-neutralizing antibody or IgG every other day (Figure 5D). After 2 weeks, retrieval of normal CD45^+^ cells and proportions of CTV^+^ cells were affected as described above in the IgG-treated group, but STC1 neutralization partially rescued these effects (Figure 5, E and F). Interestingly, neutralizing STC1 had no effect on AML cell growth (Figure 5E). These data suggest that aberrant STC1 secretion negatively regulates HSPC cycle activity in vivo.

### HIF-1α stabilization induces STC1 expression in MSCs.

**Figure 6:**
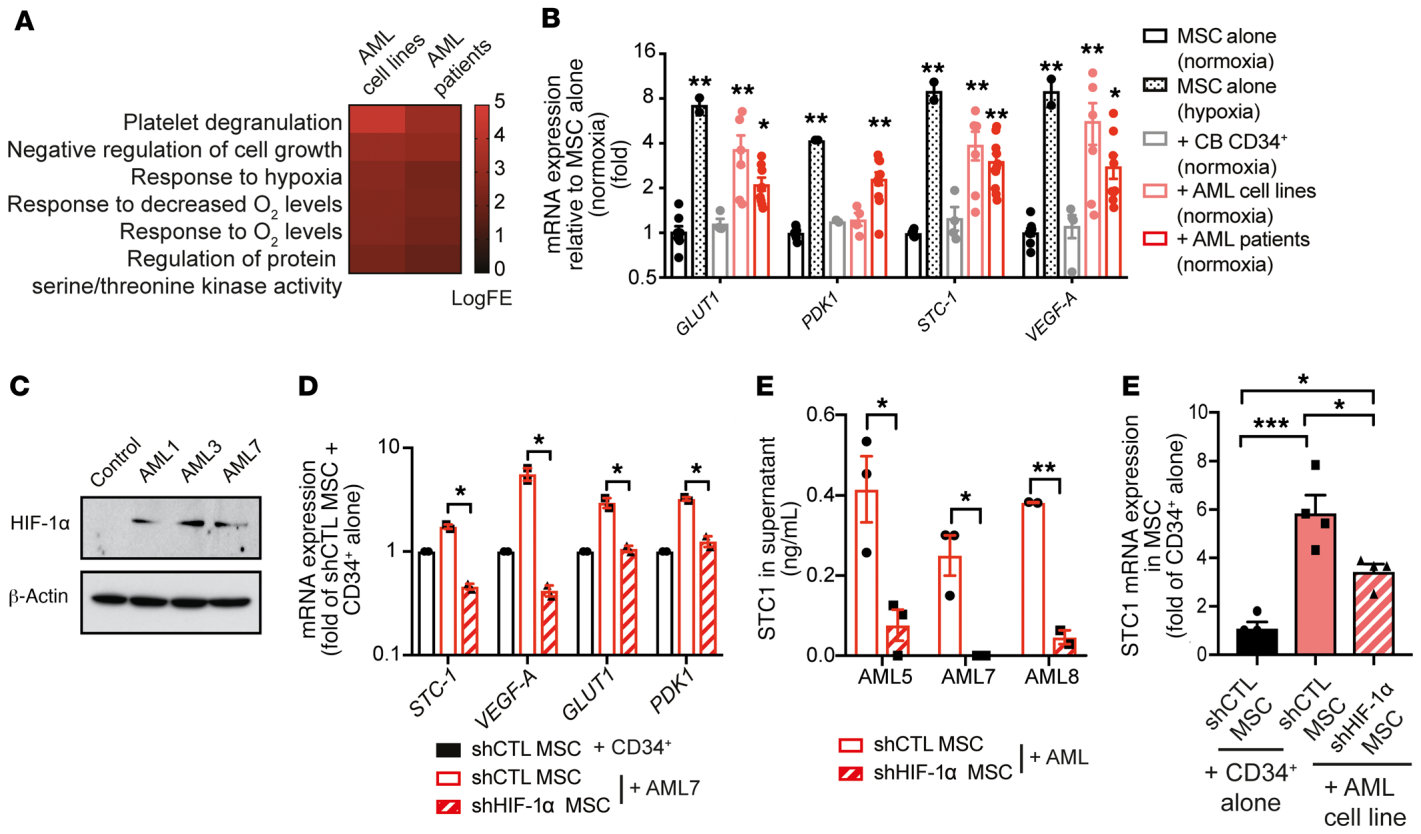
HIF-1α stabilization in MSCs induces STC1 secretion in AML. (**A**) Common gene ontology of upregulated genes from MSCs cocultured with AML cell lines or patient samples. logFE, logarithmic fold expression. (**B**) mRNA expression of hypoxia-regulated genes in MSCs. Data were obtained from 2–9 measurements of MSCs cultured alone at 20% O_2_ (normoxia) (*n* = 9) or 3% O_2_ (hypoxia) (*n* = 2), or cocultured with CB CD34^+^ cells (*n* = 4) or AML cell lines (*n* = 3)/patient samples (*n* = 4) for 5 days at 20% O_2_; measured by qPCR and normalized to MSCs (normoxia). AML patient samples: AML6–9. Measurements from AML cell lines, *n* = 3–4. (**C**) Immunoblot of HIF-1α and β-actin (loading control) of whole cell lysate of MSCs cultured alone or with AML patient samples. AML1, -3, and -7. (**D**–**F**) MSCs were lentivirally transduced to express shRNA against dsRed fluorescent protein (RFP; shCTL) or an shHIF-1α construct. (**D**) mRNA expression of hypoxia-regulated genes in cocultured MSCs normalized to shCTL-MSCs + CD34^+^ cells alone. AML7. *n* =2. (**E**) STC1 secretion in supernatant of MSCs + CD34^+^ cells alone or +AML5, -7, and -8. *n* = 3. (**F**) STC1 mRNA expression in transduced MSCs sorted from pooled scaffolds with CB CD34^+^ alone or +AML cell line U937. Four mice/group with 2 scaffolds per mouse. Data are presented as mean ± SEM, except in **D**, which shows mean ± SD. *P < 0.05, **P < 0.01, ***P < 0.001 by Kruskal-Wallis test with Dunn’s test (**A**), 2-tailed Student *t* test corrected with the Holm-Šidák method (**D** and **E**), and ANOVA with Tukey’s test (**F**).

STC1 expression is regulated by several pathways, including p53 (58), NF-κB (59), and HIF-1α (60), that have been implicated in AML (61–63). Gene ontology analysis of differentially expressed genes in MS-5 cells cocultured with AML suggested hypoxia signaling as a common upregulated pathway (Figure 6A). To test whether AML could upregulate several known hypoxia-regulated genes (*GLUT1*, *PDK1*, *VEGFA*) including *STC1*, we compared MSCs cultured under normoxia and hypoxia and MSCs cocultured with CB-derived CD34^+^ cells or AML in normoxic condition. Interestingly, coculture of MSCs with AML induced a transcriptional response similar to that of MSCs under hypoxic conditions (Figure 6B) and increased the stabilization of HIF-1α in MSCs (Figure 6C). Abrogation of HIF-1α via shRNA negated the upregulation of *STC1* and other hypoxia-response genes in response to AML coculture at the mRNA level (Figure 6D and Supplemental Figure 5A). Additionally, shHIF-1α MSCs produced less STC1 in the cell culture supernatant (Figure 6E and Supplemental Figure 5B) and expressed reduced levels of *STC1* mRNA in scaffolds with AML (Figure 6F). These data implicate HIF-1α stabilization in MSCs as an initiator of STC1 secretion in response to AML.

### HIF-1α regulates HSPC engraftment and proliferation.

**Figure 7:**
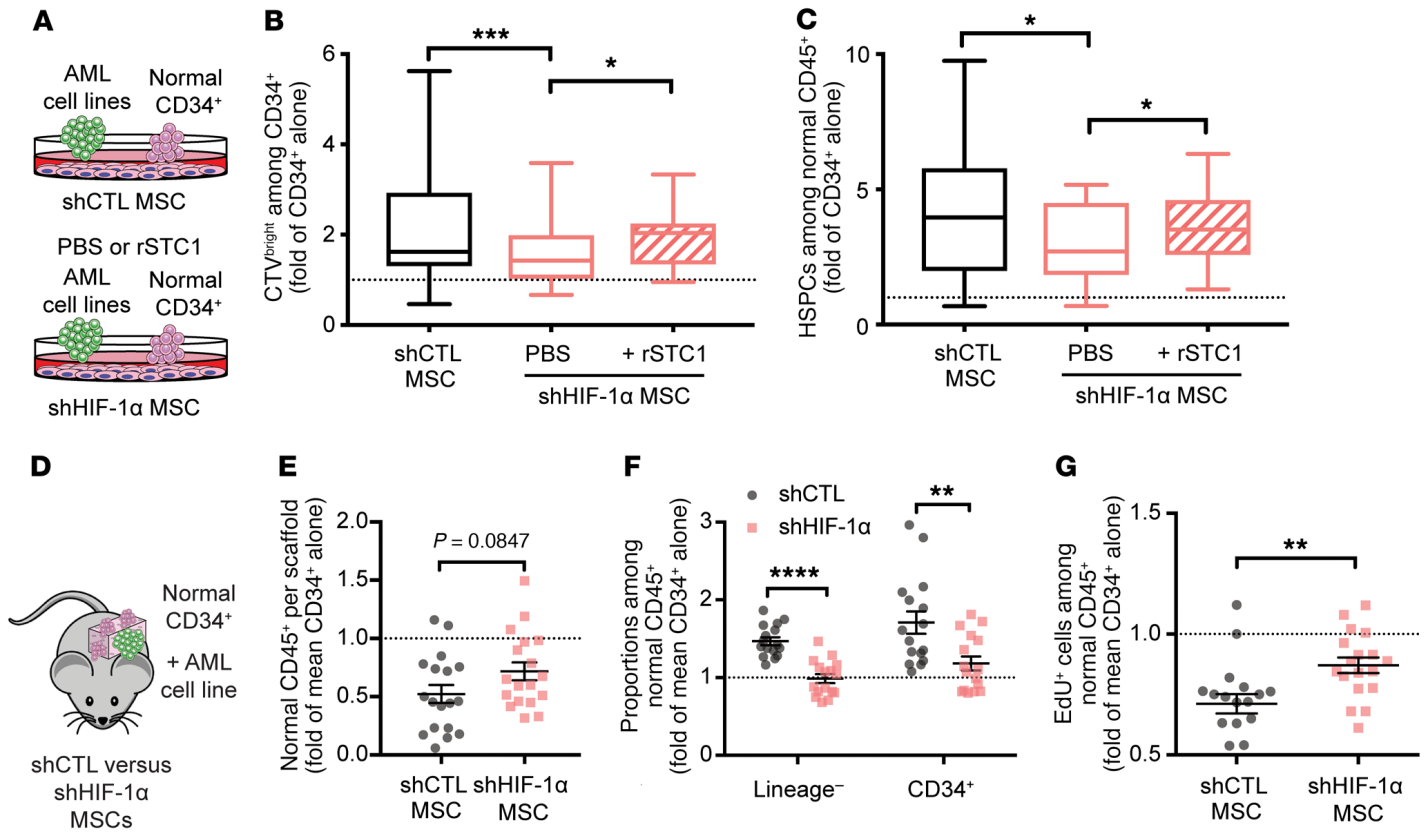
HIF-1α knockdown in MSCs partially rescues HSPC proliferation in AML. (**A**–**C**) MSCs were lentivirally transduced to express the shCTL or shHIF-1α construct and cocultured with CD34^+^ cells alone or AML cell lines with PBS or rSTC1. CD34^+^ cells alone were used as control and reference for normalization. Data are presented as box-and-whisker plots, with bounds from 25th to 75th percentile, median line, and whiskers ranging from smallest to largest values of 3 measurements per AML cell line (KG1A, OCI-AML3, U937, ML1, MOLM-13). (**B**) CTV^bright^ cells among CD34^+^ cells. (**C**) Proportions of normal HSPCs among normal CD45^+^ cells. (**D**–**F**) MSCs were lentivirally transduced to express shRNA against RFP (shCTL) or shHIF-1α construct, and seeded in scaffolds before the injection of normal CD34^+^ cells alone or with the AML cell line U937. Each dot represents data from 1 scaffold. Five mice/group with 2–4 scaffolds per mouse. (**E**) Cell counts of total normal CD45^+^ cells. (**F**) Proportions of lineage^–^CD34^+^ cells among normal CD45^+^ cells. (**G**) Proportions of EdU^+^ cells among normal CD45^+^ cells. Data are presented as mean ± SEM. *P < 0.05, **P < 0.01, ***P < 0.001, ****P < 0.0001 by Friedman’s test with Dunn’s test (**B** and **C**) or Mann-Whitney *U* test (**E**–**G**).

We next abrogated HIF-1α via shRNA in MSCs, and compared cell cycle activity and phenotypic retention of primitive HSPCs in coculture with AML cell lines (Figure 7A). Both retention of CTV^bright^ among CD34^+^ cells and proportions of HSPCs were reduced with shHIF-1α MSCs. This was partially rescued by re-supplementation of rSTC1 in the culture (Figure 7, B and C). These data suggest that HIF-1α stabilization promotes HSPC quiescence in part by secretion of STC1.

Last, we investigated whether HIF-1α was also responsible for the reduced engraftment of CD34^+^ cells in leukemic scaffolds. Control shRNA and shHIF-1α MSC scaffolds were coinjected with CD34^+^ cells and the AML cell line U937 and retrieved from NSG-S mice after 14 days (Figure 7D). shHIF-1α increased the production of normal CD45^+^ cells (Figure 7E) by rescuing the accumulation of undifferentiated cells (decrease in lineage^–^ and CD34^+^ cells) (Figure 7F), which was accompanied by increased cell cycle activity, measured by EdU incorporation (Figure 7G). These data suggest that in vivo, stabilization of HIF-1α in MSCs is required for suppression of CD34^+^ proliferation in the presence of AML.

## Discussion

Severe cytopenias are a prominent clinical feature of AML independent of blast percentage in the BM, and major contributors to morbidity and mortality in patients with leukemia (38). Data from AML patients, murine AML models, and xenotransplantation models have demonstrated that HSPCs are preserved in AML, but nonleukemic progenitors are depleted (40, 42). Normal HSPC generation is reduced in AML patients, and they become increasingly quiescent (40–42). In agreement with this, three-quarters of AML patients experience rapid reversal of marrow failure after chemotherapy treatment (64), indicating that the HSPC pool remains mostly intact and that cell cycle inhibition of HSPCs is reversible (65). However, the process by which normal HSCs and HPCs are influenced during leukemic cell infiltration is still poorly understood, involving several potentially interconnected pathways. Indirectly, AML cells — by altering BM niche components (30, 66) via exosomes (45, 46, 67) or TGF-β1 release (30, 31, 43) and/or by modifying the metabolic milieu (28, 68) — affect HSC proliferation and/or mobilization out of the niche. On the other hand, direct routes affecting erythrocyte and megakaryocyte progenitors via CCL3 (69) and thrombopoietin scavenging (38) may also exist.

In this study, we generated 2 humanized mesenchymal niche model systems, and in agreement with our previous report on AML patient samples (40), we observed a retardation of HSPC differentiation and cell cycle activity. Functional validations, including the current gold standard long-term repopulating assay, confirmed a reversible preservation of HSPCs. These experiments demonstrate that events mimicking BM failure at the HSPC level can be recapitulated with humanized mesenchymal niche model systems both in vivo and ex vivo.

We show that HSPC suppression is multifactorial and potentiated via the mesenchymal niche. Indeed, factors secreted from the MSCs in contact with leukemic cells play major roles in HSPC malfunction, although for certain AML samples, direct and contact-dependent effects were also observed.

Using a transcriptomic approach, we aimed to identify common niche-derived factors and pathways. Besides STC1, we detected previously annotated regulators of HSPCs, but we focused on STC1 for its potent role in solid cancers (70–74) and as an MSC-derived regulator of apoptosis (75), metabolism (73), and redox homeostasis (73, 75). STC1 was originally described as global calcium regulator generated in the kidney of bony fish (76), but murine knockout did not alter the phenotype (77, 78). On the other hand, overexpression of STC1 caused dwarfism and bone defects (79, 80), which may imply that aberrant concentrations, as reported for cancer, might induce additional effects not seen in the knockout models. Furthermore, the receptor(s) and structure of STC1 are unknown, which hampers detailed mechanistic analysis.

Our functional and transcriptomic analysis suggested that addition of recombinant hSTC1 retained HSPC quiescence and stemness. Further analysis of the role of MSC-secreted STC1 factor in human HSC regulation might provide additional insights into adult HSC biology in the context of disease, stress, and aging.

We further confirmed elevated levels of STC1 in BM and PB plasma of AML compared with healthy control samples and demonstrated that STC1 levels decreased during remission. It might be of interest to analyze the potential correlation between STC1 level in AML samples and cytopenic markers in a larger patient cohort.

Upregulation of hypoxia-regulated factors occurred rapidly in MSCs when cocultured with AML, but this is not maintained after leukemia cells are withdrawn from the culture, potentially explaining why STC1 and HIF-1α have not been identified in AML patient–derived MSCs (31, 35). Hypoxia was reported to preserve HSC stemness (17, 19), but there have been contradictory results about the role of HIF-1α. Deletion of HIF-1α in both hematopoietic cells and MSCs with Mx-1 (81) but not in hematopoietic cells alone with vav-1 (82) compromised HSC preservation. Furthermore, murine MSCs expressing HIF-1α attenuate HSPC differentiation, and inhibition of HIF-1α permits cell cycle activation after injury (22). We speculate now that the reported effect was mediated by STC1. Our experiments further confirm that HIF-1α functions as a proliferation repressor in the human context, mediated via its downstream effector STC1.

In summary, our study provides not only humanized models to study the crosstalk among HSPCs, leukemia, and their MSC niche, but also a mechanistic insight into the reversible suppression of HSCs in leukemic hosts, via remodeling of the MSC niche. Clinically, HIF-1α inhibition or more specifically STC1 neutralization represents a possible route to promote residual normal hematopoiesis and thus alleviate cytopenia in AML. This may be particularly relevant for older adults not determined to be tolerant of intensive therapy or for patients with adverse cytogenetic factors for whom chemotherapy is unlikely to produce a long-lasting response.

## Methods

Further information can be found in Supplemental Methods and Supplemental Tables.

### Cell lines.

HL60, KG-1A, ML1, U937, KG1, OCI-AML3, MOLM-13, NB4, Fujioka-1, and Kasumi-1 AML cell lines were originally obtained from ATCC and maintained by the Francis Crick Institute Cell Bank. Each cell line was validated by short tandem repeat (STR) profiling using the PowerPlex 16HS system (Promega). All cells were grown as suggested by the suppliers at 20% O_2_ (or 3% O_2_ for hypoxia conditions). The murine stroma cell line (MS-5) was purchased from LGC Standards. For some experiments, AML cell lines were transduced with lentivirus containing EGFP or a iCASP9 (51).

### AML patient samples.

AML patient samples (PBMCs and/or BM) were collected at diagnosis from patients at St Bartholomew’s Hospital, London. Plasma (from PB or BM) was obtained from patients at diagnosis or remission at the Royal Marsden Hospital. Details of patient samples are provided in Supplemental Tables 1 and 2.

### Umbilical CB.

Umbilical CB (UCB) samples were obtained after normal full-term births. Mononuclear cells (MNCs) from 2 to 5 UCB collections were pooled and purified by Ficoll-Paque density centrifugation (GE Healthcare Life Sciences), followed by ammonium chloride red cell lysis. Density-separated CB MNCs were magnetically sorted for CD34 positivity via the EasySep Human CD34 Positive Selection Kit (STEMCELL Technologies) according to the manufacturer’s instructions.

### Human BM–derived MSCs.

Primary hMSCs were purchased (Lonza) or provided by Christine Dosquet (Paris Diderot University, Paris, France) from human BM obtained during orthopedic surgery. hMSCs were grown in α-MEM and 10% hMSC-specific FBS (Gibco-Life Technologies) and used at low passages (lower than 5th in all cases). A total of 3 samples were used throughout this study. The phenotype of hMSCs was hCD45^–^, hCD31^–^, hCD90^+^, hCD73^+^, and hCD105^+^.

### Culture of MSCs with primary CB CD34^+^ HSPCs/AML patient samples and AML cell lines.

All culture of HSPCs/AML patient samples was carried out on primary hMSCs unless stated otherwise. On day 0, 2 × 10^4^/cm^2^ MSCs were seeded in tissue culture–treated plates (Falcon) containing 285 μL/cm^2^ α-MEM supplemented with 10% MSC-FBS and 100 U/mL penicillin/streptomycin (all Thermo Fisher Scientific). On day 2, UCB CD34^+^ cells alone or together with AML were added to the hMSC culture at 0.4 × 10^5^ to 1 × 10^5^ CD34^+^ cells/mL and 1 × 10^5^ to 8 × 10^5^ cells/mL (AML patient samples), or 2 × 10^5^ cells/mL (AML cell line) were added in MyeloCult H5100 (STEMCELL Technologies). Fresh media top-up was performed after 4 days. AML cell lines were lentivirally transduced to express GFP to distinguish non-leukemic CB CD34^+^ cells from AML cell lines.

When CD34^+^ cells were cocultured with AML cell lines and had to be re-isolated for functional analysis, 0.4-μm polycarbonate Transwell inserts (Corning) were used to separate AML cell lines from MSCs and CD34^+^ cells. Cells in the well were grown in 500 μL media or 200 μL for the Transwell. For comparison between direct contact and Transwell, CD34^+^ cells were grown in the Transwell and MSCs and AML cell lines at the bottom of the well for 4 days.

For AML patient samples, AML cells were thawed and allowed to recover for 48 hours in the MyeloCult H5100 supplemented with IL-3, G-CSF, and TPO (each 20 ng/mL, Peprotech) at densities of 0.5 × 10^6^ to 2 × 10^6^ cells/mL. Subsequently, the medium was replaced with MyeloCult H5100 containing 20 ng/mL SCF. UCB CD34^+^ cells were sorted based on HLA-A2 mismatching between UCB and AML samples.

For STC1 neutralization experiments, 1 μg/mL blocking antibody (Santa Cruz Biotechnology) or equivalent species IgG was added at the beginning of the experiment.

### Preconditioning of MSCs with AML.

MSCs were cocultured for 5 days with AML iCasp9 cell lines, then treated with 5 nM of AP1903 (Insight Biotechnology). Twenty-four hours after the addition of AP1903, all apoptotic and dead AML cells were washed away, and CD34^+^ HSPCs were seeded at 0.04 × 10^5^ to 0.1 × 10^5^/mL.

### Cell division tracking.

Cells were suspended at 1 × 10^6^/mL in PBS with 5 μM CTV or CFSE (Thermo Fisher Scientific) and incubated for 10 minutes at 37°C. Then, cells were washed twice, and the pellet was resuspended in the desired medium.

### Ki-67 and EdU staining.

For cell cycle analysis, cells were fixed and permeabilized with a Fix/Perm kit according to the manufacturer’s instructions and stained with 2 μL anti–Ki-67–APC (BD Biosciences). Staining was performed overnight at 4°C. Cells were washed in 2% FBS/PBS and resuspended in 300 μL of 2% FBS/PBS with 1 μg/mL DAPI.

For EdU analysis, 200 μL of 10 mM EdU-PBS solution was injected i.p. 16 hours before the mice were culled. After scaffold harvest and digestion, the cells were fixed and permeabilized according to the Click-iT Plus EdU Alexa Fluor 647 flow cytometry assay kit instructions (Thermo Fisher Scientific). After fixation, cells were stored overnight at 4°C to allow any remaining PFA to evaporate. The next day, cells were stained in Alexa Fluor 647 Click-iT staining cocktail for 30 minutes at room temperature and subsequently analyzed by flow cytometry. As negative control, an aliquot of cells was stained with Click-iT staining cocktail without Alexa Fluor 647 picolyl azide.

### ELISA.

STC1 measurements in the media were conducted according to the manufacturer’s instructions using the STC1 ELISA kit (R&D Systems) in cell culture supernatant. Cell culture supernatant was processed by centrifugation at 300 *g* for 5 minutes. For plasma samples, cells were removed from plasma by centrifugation for 10 minutes at 1000–2000 *g* using a refrigerated centrifuge. The supernatant was further centrifuged for 15 minutes at 2000 *g* to deplete platelets in the plasma sample.

### Human xenografts.

NOD/SCID/IL2rγ^−/−^ (NSG) mice were originally a gift from Leonard Shultz (The Jackson Laboratory, Bar Harbor, Maine, USA) and since then have been bred at the Francis Crick Institute Biological Resource facility.

Seven days before transplantation, mice were put on acidified water. Twenty-four hours before transplantation, 8- to 12-week-old mice were sublethally irradiated with 3.75 Gy from a ^137^Cs source (IBL 637 Gamma Irradiator).

For primary engraftment, 30,000–45,000 purified CD34^+^ cells and for secondary engraftment, 2 × 10^6^ purified hCD45^+^ cells (isolated with a human/mouse chimera isolation kit from STEMCELL Technologies) were injected intravenously per mouse.

Engraftment of human cells in the murine BM was assessed 12 weeks after injection. Mice were sacrificed by cervical dislocation and tibia, femur, and ilium dissected bilaterally. BM was harvested by crushing with mortar and pestle, and each mouse was processed individually. Remaining erythrocytes were lysed with 2–3 mL ammonium chloride for 3 minutes at room temperature and remaining MNCs pelleted in 2% FBS/PBS before analysis by FACS.

### Humanized scaffold.

All the experimental procedures were performed as described by Abarrategi et al. (14). Briefly, Gelfoam gelatin sponges (2 cm × 6 cm × 7 mm) (Pfizer) were cut into 24 equally sized squares, washed once with 70% ethanol and twice with PBS. MSCs were resuspended in α-MEM with 10% FBS at 1 × 10^6^ cells/mL, and 100 μL (1 × 10^5^ cells) was injected into each scaffold. The scaffolds were cultured in Nunclon Sphera low-attachment 24-well plates (Thermo Fisher Scientific) and MSCs allowed to adhere for 3–5 hours before the addition of media. Scaffolds were then cultured for 3–5 days to allow MSCs to expand. HSPCs (1 × 10^5^ CD34^+^), AML cell lines (0.1 × 10^5^); and AML patient samples (1 × 10^5^ to 5 × 10^5^ MNCs, T cell–depleted) were injected into these MSC-seeded scaffolds; and medium was replaced with MyeloCult H5100 supplemented with cytokines (20 ng/mL G-CSF, 20 ng/mL IL-3, and 20 ng/mL TPO from PeproTech) when AML patient samples were used. After seeding of the cells in the scaffold, the scaffolds were implanted subcutaneously into nonconditioned NSG mice (maximum 4 scaffolds/mouse).

At different end points, scaffolds were harvested and digested in an HBSS digestion solution containing 2% FBS, DNAse I (10 μg/mL), collagenase (1 mg/mL), dispase II (5 mg/mL), and heparin (20 U/mL) at 37°C for 1.5 hours. The reaction was stopped by addition of 3 times the volume of MSC media, and cells were washed twice before resuspension of the pellet and analysis of the cells by FACS.

### Microarray data.

Microarray analysis was performed on MS-5 cells cocultured for 5 days with 5 AML cells lines, 2 primary patient samples, or CB CD34^+^ cells. Microarray data were analyzed using limma and affy packages in the R environment (R version 3.5.1). Raw CELL files were uploaded with the ReadAffy function, and robust multiarray averaging (RMA) normalization was then performed. A differentially expression statistical analysis was consequently performed in which we set up the experimental design, including conditions and batch effect as factors. Differentially expressed genes were then obtained by contrasting the different conditions using the makeContrasts function. Given the linear model fit, the statistics were computed by empirical Bayes moderation of the standard errors with the eBayes function.

### RNA-Seq data.

RNA-Seq analysis was performed on CB-derived CD34^+^CD38^–^ cells cultured for 48 hours with or without rSTC1. Raw reads were quality and adapter trimmed using cutadapt 1.9.1 (83) before alignment. Reads were then aligned and quantified using RSEM 1.3.0/STAR 2.5.2 (84, 85) against the human genome GRCh38 and annotation release 86, both from Ensembl. Fragments per kilobase million (FPKM) and transcripts per kilobase million (TPM) values were also generated using RSEM/STAR. RNA-Seq data were analyzed using the DESeq2 package in the R environment. Raw counts were uploaded, and a DESeq object was created taking into account conditions and pool of donors as batch effect on the experimental design. Normalization was then performed using the DESeq function. Regularized log transformation was done with the rlog function. Pairwise comparisons were then performed with the contrast function, from which genes differentially expressed between different conditions were determined.

GSEA (ref. 86, version 3.0) was performed using Preranked analysis. The gene lists were ranked using the Wald statistic, and the program was run with the classic scoring scheme. The gene sets used (C1–C7 and H) were in version 6.2.

### Data availability.

Microarray and RNA-Seq data have been deposited in the NCBI’s Gene Expression Omnibus database (GEO GSE136883, MS-5 microarray; GSE136884, RNA-Seq of HSPCs with or without rSTC1). Reagents and resources used in this article are described in Supplemental Methods.

### Statistics.

Results are shown as mean ± SEM unless stated otherwise. Box-and-whisker plots show bounds from 25th to 75th percentile, median line, and whiskers ranging from smallest to largest values All statistical analyses were performed using GraphPad Prism software. A *P* value less than 0.05 was considered significant. Normal distribution was assessed by D’Agostino-Pearson normality test for groups with 8 or more and Shapiro-Wilk test for 3–7 samples. For data sets composed of heterogeneous groups (AML cell lines or patient samples combined into 1 group), nonparametric tests were performed. For 2 paired groups, we used Wilcoxon’s matched-pairs signed-rank test; for 2 unpaired groups, Mann-Whitney *U* test; for 3 or more paired groups, Friedman’s test with Dunn’s test corrected for multiple comparisons. For 3 or more unpaired groups, we used Kruskal-Wallis with Dunn’s test corrected for multiple comparisons, except for the CD34^+^-alone group and +AML cell lines/AML patient samples group ex vivo, which were compared with Mann Whitney *U* test with Bonferroni’s correction for multiple comparisons. For data sets composed of homogeneous groups (each group was an individual cell line or patient sample, vehicle versus rSTC1 or IgG versus anti-STC1), parametric tests were performed. For 2 paired groups, we used paired 2-tailed Student’s *t* test; for 2 unpaired groups, 2-tailed Student’s *t* test; for 2 or more unpaired groups, ANOVA. Multiple comparisons were corrected with Dunn’s test for Kruskal-Wallis and Friedman’s tests, Holm-Šidák for multiple Student’s *t* tests and Mann-Whitney *U* tests, and Tukey’s for ANOVA.

### Study approval.

AML samples, healthy BM samples, and UCB samples were collected after obtaining written informed consent. This study was approved by the East London and the City Research Ethics Committee in accordance with the Declaration of Helsinki (REC 06/Q0604/110). Human BM MSCs were provided by Christine Dosquet (Paris Diderot University) from human BM obtained during orthopedic surgery (under ethical approval 10-038 from IRB00006477, Hôpital Saint-Louis, Paris, France). All animal experiments were performed at the Francis Crick Institute in accordance with UK Home Office and Crick guidelines and were undertaken under Home Office project license PLL 70/8904.

## Author contributions

AW, AH, KRP, and DB conceptualized the study. AW, KRP, AA, and AH developed the methodology. AW, AH, MGA, FMM, NB, AA, KRP, and DB analyzed and interpreted results. AW and DB wrote the original draft of the manuscript. AW, JG, JF, DT, and DB reviewed and edited the manuscript. JF, DT, and DB acquired funding. DB, DT, and JG provided resources. AH, KRP, and DB supervised the study.

## Supplementary Material

Supplemental data

## Version 1 05/04/2020

Electronic publication

## Version 2 06/01/2020

Print issue publication
