# Supplementary material for: Mesenchymal niche remodeling impairs hematopoiesis via stanniocalcin 1 in acute myeloid leukemia
Source: J Clin Invest. 2020 May 4;130(6):3038–50. doi: 10.1172/JCI133187 (PMC7260026; doi:10.1172/JCI133187)
Supplement: Supplemental data [file jci-130-133187-s092.pdf]

Supplementary Figures

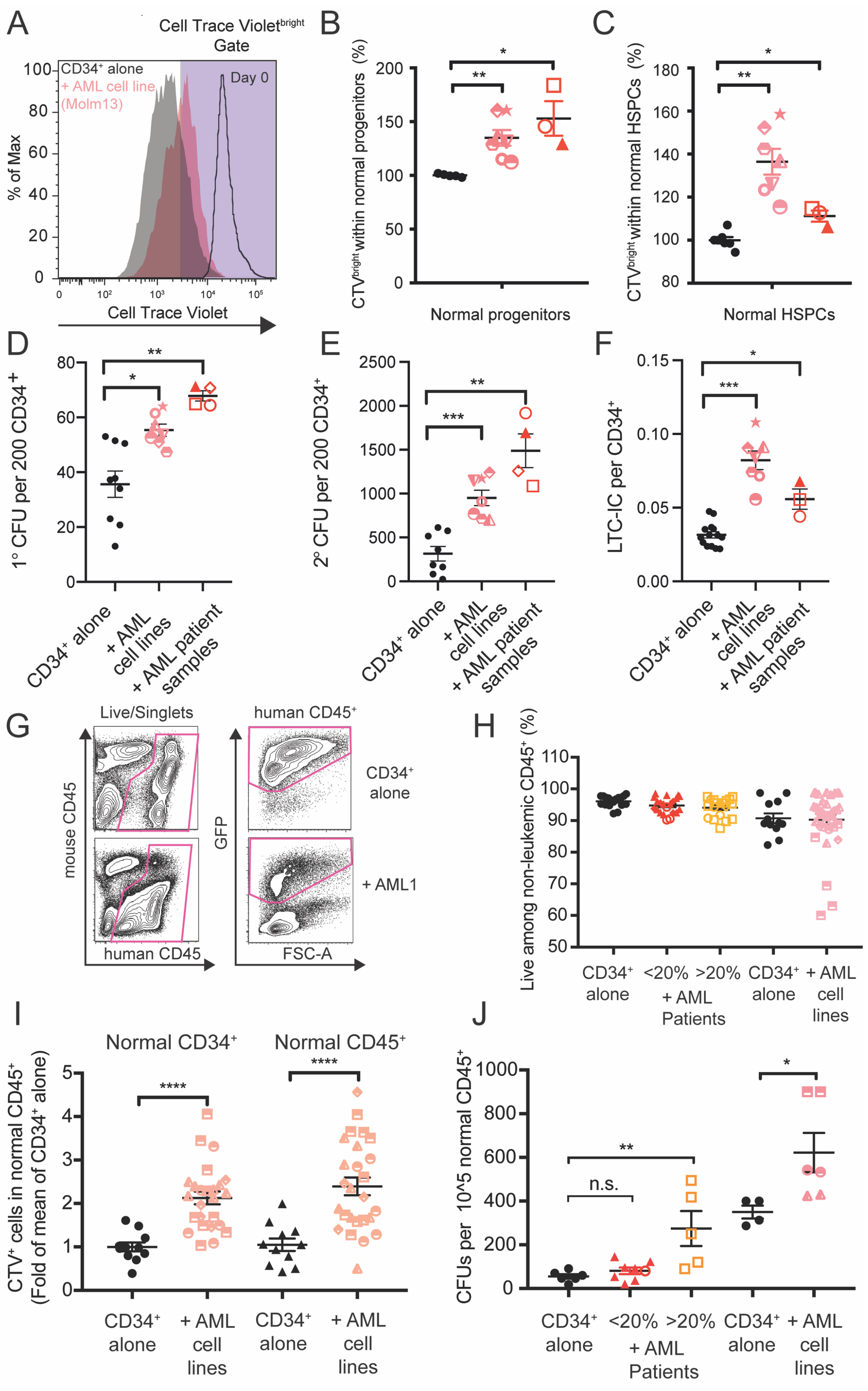

Supplementary Figure 1. (A-F) $CD34^+$ cells co-cultured with MSCs alone ($CD34^+$ alone) (n=4-7), + AML cell lines (n=7) or primary samples (n = 3-4). After 4 days of co-culture, $CD34^+$ cells were plated for CFU or LTC-IC assays. **(A)** Representative flow cytometry plot of $CD34^+$ cells before and after co-culture with MSCs alone or + AML cell line MOLM-13 and gating strategy to analyze the proliferation based on Cell Trace Violet (CTV) retention. **(B-C)** Quantification of CTV^{bright} retaining cells among normal progenitors ($CD34^+CD38^+$) **(B)** and HSPCs ($CD34^+CD38^-$) **(C)** from $CD34^+$ co-cultured with MSC alone or AML cell lines (n=7) and patient samples. AML1-4. **(D-F)** Sorted $CD34^+$ cells after 4 days co-culture with MSCs alone, or with AML cell lines (n=3-7) or AML patient samples (n=3) were plated for CFU or LTC-IC assays or implanted into NSG mice. AML patients: AML1-3 **(D)** Primary and **(E)** secondary CFU results per 200 $CD34^+$ cells. **(F)** Frequency of LTC-IC. **(G)** Flow cytometry gating strategy to identify human hematopoietic engraftment and distinguish normal from leukemic cells from $CD34^+$ alone (top) and + AML patient scaffolds (bottom), respectively. **(H)** Percentage of viable non-leukemic human $CD45^+$ cells per scaffold. AML1-5. 12-35 scaffolds in 5-9 mice/group **(I)** Quantification of CTV^+ cells among normal HSPCs ($CD34^+$) and non-leukemic human $CD45^+$ from scaffolds of $CD34^+$ alone (n=7) or + AML cell lines (n=4). 12-27 scaffolds in 6-9 mice/group **(J)** Primary colony forming ability of non-leukemic FACS-sorted cells from $CD34^+$ alone or + AML scaffolds. AML cell lines (n=3) and AML patients (n=4)

Each symbol represents an AML cell line/patient sample and data is presented as mean ± SEM.. n.s. non-significant, *P < 0.05; **P < 0.01; ***P < 0.001, ****P < 0.0001 by Mann-Whitney test **(B-F, I)** and Krustal-Wallis with Dunn’s **(J)**.

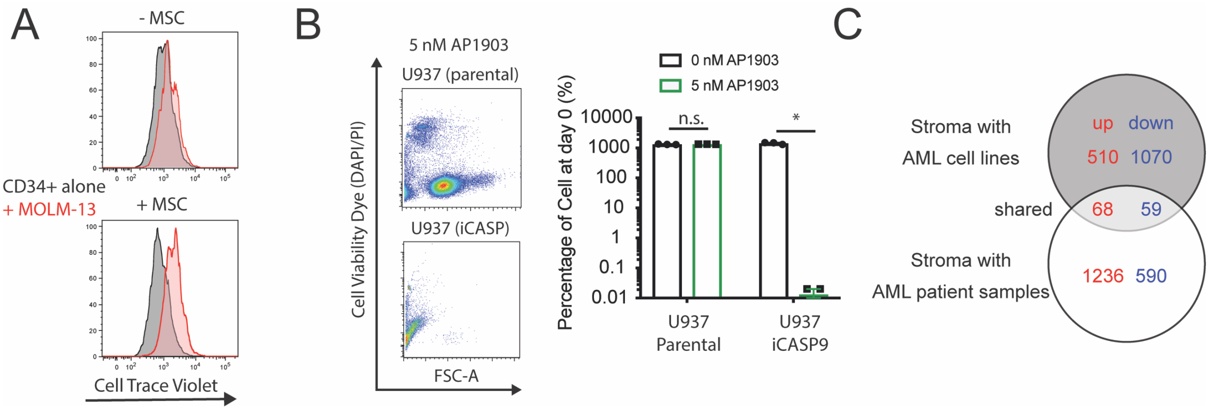

Supplementary Figure 2 (A) CD34⁺ cells were cultured alone or with AML cell lines (n=4) with (+ MSC) and without (-MSC) MSCs. Representative flow cytometry plots with CD34⁺ alone (grey) and + MOLM-13 (red). **(B)** Parental and iCASP9 AML cell lines were exposed for 4 days to AP1903 or vehicle. (Left panel) Flow cytometry plot of parental and iCASP9 AML cell line U937. (Right panel) Cell count normalized to day 0. **(C)** Differentially up- or downregulated genes with log fold-change (LogFC) <-1 or >1 in MS-5 with AML cell lines, or AML patient samples or shared between both groups Data is presented as mean ± SEM of n=3. n.s. non-significant, *P < 0.05; **P < 0.01; ***P < 0.001, ****P < 0.0001 by 2-tailed Student T-test

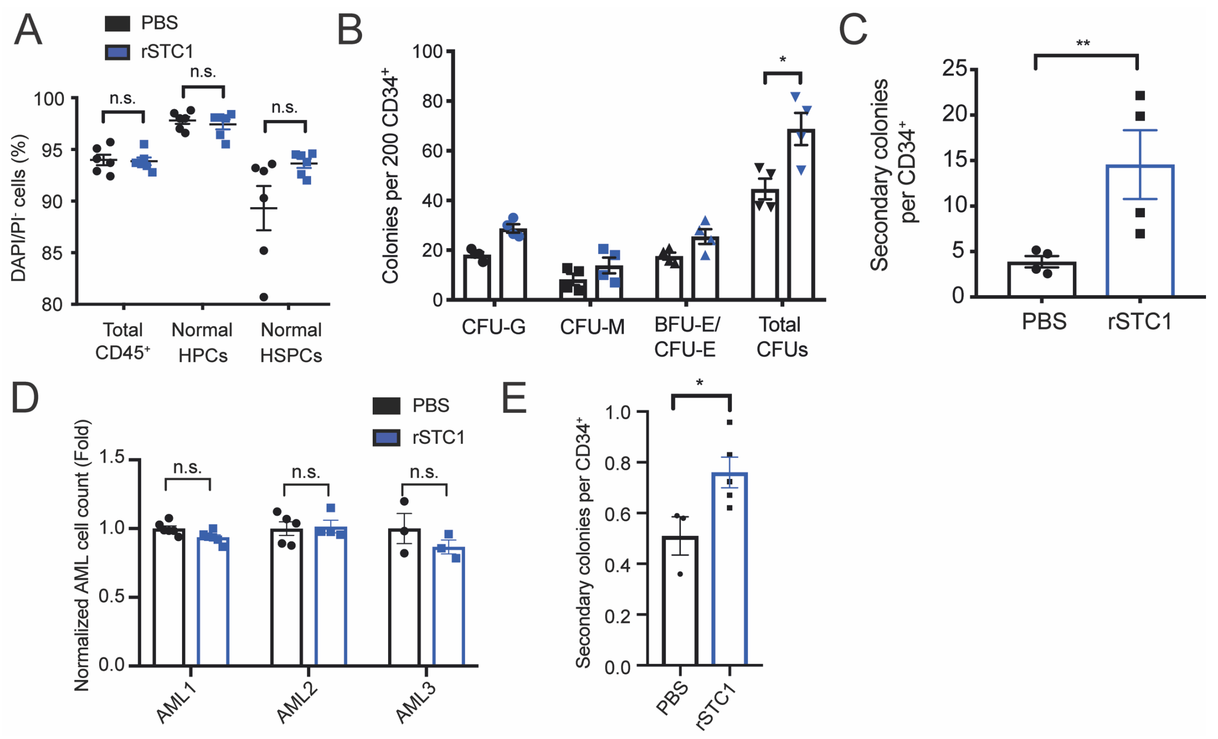

Supplementary Figure 3 (A-D) Normal CB CD34$^+$ or AML patient samples were co-cultured on MSCs and either PBS or rSTC1 was added for 5 days. **(A)** Quantification of viable DAPI$^-$/PI$^-$ cells **(B-C)** Normal CD34$^+$ cells were sorted after 5 days and plated for CFC assay **(B)** Primary CFUs per 200 CD34$^+$ cells; **(C)** Total secondary CFUs. **(D)** Total leukemic cell count normalized to PBS control. **(E)** Normal BM CD34$^+$ co-cultured on MSCs and either PBS or rSTC1 was added for 5 days. Secondary CFUs per CD34$^+$ in BM CD34$^+$.

Data is presented as mean ± SEM of n=3-6. n.s. non-significant, *P < 0.05; **P < 0.01; ***P < 0.001, ****P < 0.0001 by 2-tailed student t-test

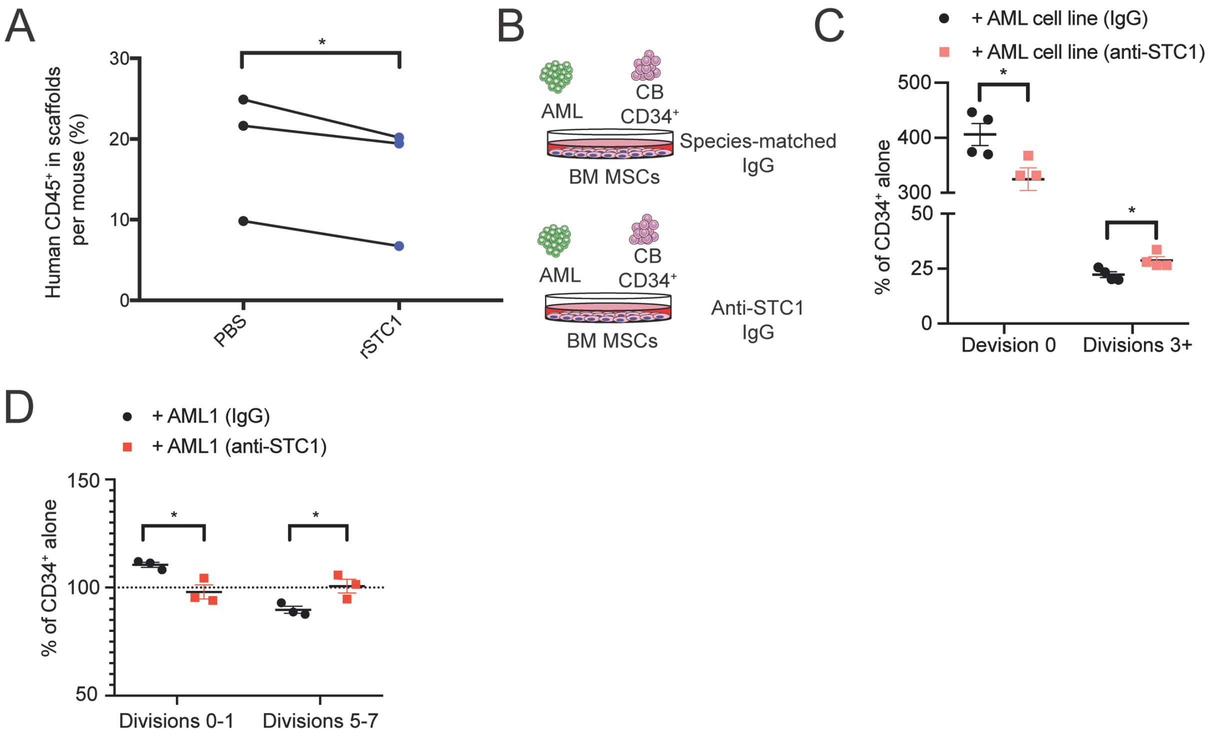

Supplementary Figure 4 (A) MSC scaffolds with normal CD34$^+$ were implanted into NSG-S mice. rSTC1 was injected every other day for 10 days. Engraftment of human CD45$^+$ cells in scaffolds per mouse. Paired 2-tailed Student T-test. **(B-D)** CD34$^+$ cells were co-cultured with MSCs alone or + AML cell line U937 (C) or + AML1 **(D)** and supplemented with Anti-STC1 antibody or species-matched IgG. **(C-D)** Proportions of cells undergone n divisions relative to CD34+ alone.

Data is presented as mean ± SEM of n=3-4 n.s. non-significant, *P < 0.05; **P < 0.01; ***P < 0.001, ****P < 0.0001 by 2-tailed student t-test

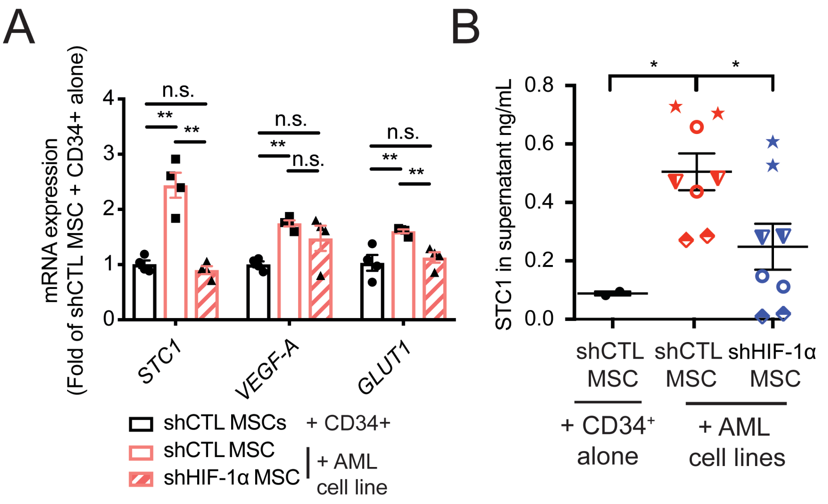

Supplementary Figure 5 (A-B) MSCs were lentivirally transduced to express shRNA against RFP (shCTL) or shHIF-1α (shHIF-1α) construct. **(A)** mRNA expression of hypoxia regulated genes in MSCs after co-culture with CB $CD34^+$ (n=4) or AML cell line U937 (n=4) and normalized to shCTL-MSC + $CD34^+$ alone. **(B)** STC1 secretion in supernatant of MSCs + $CD34^+$ alone (n=4) or + AML cell lines (n=4).

Data is presented as mean ± SEM n.s. non-significant, *P < 0.05; **P < 0.01; ***P < 0.001, ****P < 0.0001 by ANOVA with Tukey test **(A)** and Friedman test with Dunn's test **(B)**.

Supplementary Materials and Methods

Lentivirus transduction

MSC

MSCs were seeded at $2.5x10^4$/cm2 in a 12-well dish in 400 µL of media at a multiplicity of infection (MOI) of 10 and incubated at 37° C. After 16h the virus is removed and the MSCs are trypsinized and seeded. All co-cultured and scaffolds were started 4 days after MSC transduction. Transduction efficiency was generally >80 %.

HSPCs

$CD34^+$ HSPCs were resuspended at $0.2\text{-}0.5x10^6$/mL Stem span SFEM II (StemCell Technologies, Vancouver, Canada) containing SCF 100 ng/ml, Flt3L 100 ng/ml, IL6 10 ng/ml, IL3 10 ng/ml, G-CSF 25 ng/ml (all cytokines from Peprotech, London, UK) and 100 U/ml P/S and pre-stimulated for 8h before adding lentivirus at MOI of 30. After 16h the cells are washed with PBS and resuspended in Stem span SFEM II containing SCF 300 ng/ml, Flt3L 300 ng/ml and TPO 20 ng/ml. After another 2 days the GFP^+ HSPCs were FACS-sorted.

Generation of Lentivirus vectors

The generation and validation of lentivirus vector against HIF-1α (Sh-HIF1) is described in our previous publication (20). Briefly, H1-shRNA sequences were subcloned in the lentivirus (pTripΔU3Ef1α-EGFPMCSΔU3) that contains the enhanced GFP (eGFP) gene under the control of the EF1α promoter. An shRNA directed against the dsRed fluorescent protein (RFP) was used as a control (shCTL).

Lentivirus vectors against STC1 were purchased from vectorbuilder based on U6-shRNA sequences with eGFP selection marker. As control an shRNA directed against LacZ was used. shRNA sequences are listed in **Table S2.**

Generation of AML expressing inducible caspase 9 fragment

A plasmid with the MSCV.IRES.GFP retrovirus backbone expressing a product of FKBP1A fused with a Ser-Gly-Gly-Gly-Ser linker to the small and large subunit of the caspase 9 gene (originally deposited by David Spencer) was obtained from Addgene. Retrovirus production was carried out identically to the lentivirus production with the exception that retrovirus packaging constructs pHIT60 (expressing gag-pol) and pVSV-g (expressing VSV-G) were used (both a generous gift from Jonathan Stoye). AML cell lines were transduced at MOI 10 and sorted four days after based on GFP^+ expression (named AML iCasp9).

Colony forming unit assay

To perform colony forming unit assays, $CD34^+$ cells were plated in the methylcellulose media MethoCult H4434 (StemCell Technologies, Vancouver, Canada). Colonies were scored according to their morphology 14 days following plating using an inverted microscope. Colonies were harvested by washing with PBS and re-plated in secondary assays for an additional 14 days.

Long term colony initiation cell – Assay (LTC-IC)

As a feeder-layer MS-5 were seeded at $2x10^4/cm^2$ on day 0 in a 12-well dish. On day 2 the plate was irradiated at 7.5 grey. 4,000 $CD34^+$ HSPCs are suspended in 1 ml of Myelocult H5100 (Stem cell technologies, Vancouver, Canada). The media from the irradiated feeder-layer plate is removed and replaced with 1 ml of the HSPC

suspension per well. 500 µl of media was replaced per week. After 5 /6 weeks, all cells are collected and FACS sorted using a surface marker staining for human CD45 and murine SCA-1 to discriminate MS-5 (Sca-1^{+}CD45^{-}) and hematopoietic cells (Sca-1^{-} CD45^{+}). 2.2×10^{4} hematopoietic cells were resuspended in 1.1 ml of methylcellulose (Methocult H4434 Stem Cell Technologies, Vancouver, Canada) containing SCF, GM-CSF, IL3 and EPO at manufacturer's concentration. One ml of the suspension was transferred to one of the 6 inner wells of a 12-well dish. The outer wells were filled with dH2O to prevent the methylcellulose from drying. Colonies are scored after 14 days. The number of colonies from 2×10^{4} cells was used to calculate the total number of colonies from the progeny of 4,000 HSPCs. The total colony number was then divided by the average colony output per LTC-IC which determines the number of LTC-IC per 4,000 HSPCs.

Flow cytometry analysis and cell sorting

Flow cytometry analysis was performed using a LSRII flow cytometer and LSRFortessa (BD Biosciences, Oxford, UK). Transduced cells were identified based on their GFP expression or via HLA.A2 (mismatched between UCB and primary patient samples sued). Mouse Human CD73 and CD90 was used to label primary MSC. Sorting was performed on either MoFlo XDP (Beckman Coulter), BD FACS Aria or BD Influx sorters using a 100 µm nozzle. Collection tubes contained 2% FCS/PBS and purity checks were performed to check sort quality. Human grafts in mice were assessed using hCD45, hCD3, hCD19, hCD33, hCD34, hCD38, hCD45RA, hCD90, hCD49f and mouse CD45 antibodies (see table supplementary S2).

Reverse Transcriptase and Real Time Quantitative PCR (RT-QPCR)

The direct-zol RNA purification kit (Zymo Research) was used for samples <1x10^5 cells and the RNeasy Mini kit (Qiagen RNeas, Crawley, UK) for samples between 1x10^5 - 1x10^7 cells according to the manufacturer's instructions. mRNA was reverse transcribed by Superscript III Reverse Transcriptase (Invitrogen) with an oligoDT primer (Sigma-Aldrich, Gillingham, Dorset, UK). RT-QPCR was performed on a QuantStudio™ 5/7 (Applied Biosystems) using the standard manufacturer's settings and mRNA expression was quantified with the Comparative CT Method and Ribosomal Protein L18A (RPL18A) (MSC) were used as housekeeping gene controls. The CT values used were the result of triplicates. The primers used are described in **Table Supplementary S4**.

Western blotting

Cells were lysed in RIPA buffer containing a protease and phosphatase inhibitor cocktail (all from Sigma, Dorset, UK). The protein concentration was measured with a Lowry protein assay (DC Protein Assay from BioRad). Total protein extracts (20-30 µg) were mixed with 4x Nupage Loading buffer (ThermoFisher Scientific, UK) and boiled at 96° C for 5 min. The samples were run on a denaturing precast 8-12 % bis-tris Nupage gel (ThermoFisher Scientific, UK) and transferred by wet transfer to nitrocellulose membranes. Primary antibodies were incubated ON at 4° C and secondary antibodies 1h at RT. Protein bands were visualized using an enhanced chemiluminescence visualization system (ECL Plus, Amersham Life Sciences).

Table Supplementary S1 - Primary AML patient cells used

AML ID	Karyotype	FAB	NPM	FLT3	Risk Group
AML5	Normal	Bi-phenotype	n.d.	ITD	intermediate
AML1	T(6;11)(Q27;Q23)	M5A	n.d.	n.d.	n.d.
AML2	Trisomy 8,21	n.d.	n.d.	n.d.	poor
AML3	Trisomy 13	M1	Mut	n.d.	n.d.
AML4	1q	M5A	Mut	WT	n.d.
AML6	Complex	n.d.	n.d.	n.d.	poor
AML7	Normal	M2	Mut	WT	intermediate
AML8	Normal	M1	Mut	ITD	intermediate
AML9	5q-	n.d.	n.d.	n.d.	poor
AML10	Inv(3)(Q21Q26)/t(8;13)(p21)	M0	n.d.	n.d.	n.d.
AML11	Normal	M1	n.d.	n.d.	n.d.
AML12	t(6;9)	M2	WT	ITD	poor
AML13	46,XX,t(6;9)(p23;q34)	M4	WT	ITD	poor

n.d. Not determined

Table Supplementary S2 – Primary AML patient plasma used

AML at diagnosis

Patient ID	WBC x10^9/L	PLT x 10^9/L	Neut x 10^9/L	HCT	FLT3 (ITD)	FLT3 (ITD) VAF	RUNX1	IDH1	IDH2	NPM	Plasma Origin
AML14	0.8	118	0.3	0.31	No		ND	No	No	No	BM
AML15	1.3	24	0.12	0.237	Yes	0.548	ND	No	No	No	BM
AML16	2.8	28	0.8	0.256	Yes	3.311	ND	ND	No	No	BM
AML17	0.5	51	0.07	0.199	No		ND	Yes	No	Yes	BM
AML18	10.9	40	5.53	0.332	No		ND	No	No	Yes	BM
AML19	144.8	78	1.33	0.231	Yes	0.49	ND	No	No	No	BM + PB
AML20	70.3	31	49.6	0.242	ND		ND	ND	ND	ND	BM + PB
AML21	18.3	57	4.26	0.214	No		Yes	No	No	No	BM + PB
AML22	26	30	0.26	0.332	No		ND	No	No	Yes	BM + PB
AML23	0.8	24	ND	0.222	No		ND	No	No	No	BM
AML24	0.4	91	0.12	0.302	No		ND	Yes	No	No	BM
AML25	12.3	23	1.86	0.226	failed		ND	Yes	No	Yes	BM
AML26	12.8	30	5.43	0.235	Yes	0.36	Yes	No	No	No	BM
AML27	9.1	169	1.98	0.353	No		ND	No	Yes	No	BM
AML28	1.5	33	0.22	0.249	No		ND	Yes	No	No	BM
AML29	12.8	21	0.06	0.301	No		Yes	No	No	No	BM + PB
AML30	1.2	118	0.43	0.287	No		ND	No	No	No	PB
AML31	84.3	22	ND	0.206	Yes	0.363	Yes	No	No	No	BM + PB
AML32	0.6	175	0.31	0.306	No		Yes	No	No	No	BM + PB
AML33	48.9	18	0.24	0.256	No		ND	No	No	No	BM + PB

AML34	180	25	10.08	0.218	Yes	0.87	ND	No	No	Yes	BM + PB
AML35	39.5	6	0.59	0.239	Yes	0.67	Yes	No	No	No	BM + PB
AML36	2.3	11	0.01	0.26	No		ND	No	Yes	No	PB
AML37	18.7	39	6.05	0.295	Yes	0.471	ND	No	No	No	PB
AML38	1.9	83	ND	0.22	No		ND	Yes	No	Yes	PB
AML39	27.5	25	0.81	0.211	Yes	0.33	Yes	No	No	No	PB
AML40	148.1	17	4.59	0.178	Yes	0.04	ND	No	No	Yes	PB
AML41	34	11	8.08	0.209	No		ND	No	No	No	PB
AML42	22.4	146	ND	0.308	No		ND	No	No	No	PB
AML43	88.2	24	ND	0.26	Yes	0.28	ND	No	No	No	PB
AML44	60.2	25	ND	0.157	No		ND	No	Yes	No	PB
AML45	88.4	45	16.27	0.234	No		ND	No	Yes	No	PB
AML46	48.6	36	1.22	0.255	Yes	0.75	ND	No	Yes	No	BM + PB
AML47	37.3	69	2.05	0.248	No		Yes	No	No	No	PB
AML48	43.6	16	4.05	0.337	No		No	No	No	No	PB

Paired samples

Patient ID	WBC x10^9/L	PLT x 10^9/L	Neut x 10^9/L	HCT	FLT3 (ITD)	FLT3 (ITD) VAF	RUNX1	IDH1	IDH2	NPM	Plasma Origin
AML65-D	60.5	98	0.3	0.345	No		ND	No	No	No	BM + PB
AML65-R	36.1	535	22.82	0.207							BM
AML66-D	5.5	22	2.47	0.198	Yes	0.418	ND	No	No	Yes	BM
AML66-R	7.5	401	4.99	0.336							BM
AML67-D	39.5	61	1.34	0.217	Yes	0.498	ND	No	No	No	BM + PB
AML67-R	7.3	181	3.89	0.215							BM
AML68-D	2.5	31	1.32	0.229	No		Yes	No	No	No	BM

AML68-R	12.4	97	10.63	0.354							BM
AML69-D	0.7	12	ND	0.201	No		ND	No	No	No	BM
AML69-R	2.7	302	1.53	0.306							BM

AML in remission

Patient ID	WBC x10^9/L (4 - 10)	PLT x 10^9/L (150 - 410)	Neut x 10^9/L (2 - 7)	HCT	Plasma Origin
AML49	4.6	245	2.11	0.394	BM
AML50	4	169	2.41	0.503	BM
AML51	5.6	163	4.11	0.373	BM
AML52	3.6	219	2.01	0.353	BM
AML53	3.2	280	1.29	0.343	BM
AML54	4.5	189	3.71	0.278	BM
AML55	7.8	218	5.31	0.273	BM
AML56	4.7	130	1.96	0.412	BM
AML57	2.3	109	1.19	0.274	BM
AML58	5.6	112	4.12	0.394	BM
AML59	2.7	214	1.02	0.375	BM
AML60	4.9	215	3.22	0.381	BM
AML61	3.3	117	1.74	0.311	BM
AML62	7.9	276	5.53	0.217	BM
AML63	3.8	281	1.3	0.327	BM
AML64	9	153	ND	0.258	BM

Table Supplementary S3 - Antibodies

Antigen	Species	Clone	Manufacturer	Catalogue number
β–Actin	Human	AC-15	Sigma	A1978
CD135	Human	4G8	BD	583494
CD19	Human	HIB19	BD	555412
CD3	Human	UCHT1	BD	555333
CD33	Human	WM53	BD	551378
CD34	Human	4H11	eBioscience	25-0349-42
CD34	Human	8G12	BD	347222
CD34	Human	581	BD	555824
CD38	Human	HB7	eBioscience	25-0388-42
CD45	Human	HI30	eBioscience	25-0459-42
CD45	Mouse	30-F11	eBioscience	45-0451-82
CD45RA	Human	HI100	eBioscience	47-0458-42
CD49f	Human	GoH3	BD	555736
CD51	Mouse	RMV-7	eBioscience	12-0512-83
CD73	Human	AD2	BD	563199
CD90	Human	eBio5E10	eBioscience	17-0909-42
HIF-1a	Human	54/HIF-1α	BD	610959
HLA-A2	Human	BB7.2	BD	558570
Ki67	Human	20RAJ1	eBioscience	50-5699-82
Lineage cocktail	Human	mixed	BD	340546
STC1	Human	N-15	Santa Cruz	sc-14346
STC1	Human		Biovendor	RD181095100

Table Supplementary S4 - shRNA sequences

Target	Sequence
shCTL(dsRed)	GCTCCAAGGTGTACGTGAATTCAAGAGATTCACGTACACCTTGGAGC

ShHIF-1α	GATGTTAGCTCCCTATATCCCTTCAAGAGAGGGATATAGGGAGCTAACATC

Table Supplementary S5 - RT-PCR primer sequences

Gene	Species	5' sequence	3' sequence
STC-1	Human	TGAGGCGGAGCAGAATGACT	CAGGTGGAGTTTTCCAGGCAT
STC-2	Human	GGTGGACAGAACCAAGCTCTC	CGTTTGGGTGGCTCTTGCTA
VEGF-A	Human	ATGAACTTTCTGCTGTCTTGGGT	TGGCCTTGGTGAGGTTTGATCC
RPL18A	Human	GGAGAGCACGCCATGAAG	AAGATTCGCATGCGGTAGAG
IGF-BP1	Human	TCAAAAAATGGAAGGAGCCCT	AATCCATTCTTGTTGCAGTTT
IGF-BP2	Human	CACCGGCACATGGGCAA	GAAGGCGCATGGTGGAGAT
PDK1	Human	CGGATCAGAAACCGACACA	ACTGAACATTCTGGCTGGTGA
GLUT1	Human	AACTCTTCAGCCAGGGTCCAC	CACAGTGAAGATGATGAAGAC
